# Cell cycle-linked vacuolar pH dynamics regulate amino acid homeostasis and cell growth

**DOI:** 10.1038/s42255-023-00872-1

**Published:** 2023-08-28

**Authors:** Voytek Okreglak, Rachel Ling, Maria Ingaramo, Nathaniel H. Thayer, Alfred Millett-Sikking, Daniel E. Gottschling

**Affiliations:** 1 Calico Life Sciences, LLC, South San Francisco, CA USA; 2 Present Address: Altos Labs, Redwood City, CA USA

**Keywords:** Lysosomes, Mitochondria, Homeostasis, TOR signalling, Metabolism

## Abstract

Amino acid homeostasis is critical for many cellular processes. It is well established that amino acids are compartmentalized using pH gradients generated between organelles and the cytoplasm; however, the dynamics of this partitioning has not been explored. Here we develop a highly sensitive pH reporter and find that the major amino acid storage compartment in *Saccharomyces* *cerevisiae*, the lysosome-like vacuole, alkalinizes before cell division and re-acidifies as cells divide. The vacuolar pH dynamics require the uptake of extracellular amino acids and activity of TORC1, the v-ATPase and the cycling of the vacuolar specific lipid phosphatidylinositol 3,5-bisphosphate, which is regulated by the cyclin-dependent kinase Pho85 (CDK5 in mammals). Vacuolar pH regulation enables amino acid sequestration and mobilization from the organelle, which is important for mitochondrial function, ribosome homeostasis and cell size control. Collectively, our data provide a new paradigm for the use of dynamic pH-dependent amino acid compartmentalization during cell growth/division.

## Abstract (web summary)

In this study, Okreglak et al. identify dynamic regulation of pH in the lysosome-like vacuole of growing *S.* *cerevisiae* cells and link pH dynamics in this subcellular compartment to amino acid release into the cytoplasm to meet metabolic demands during cell cycle progression.

## Main

The yeast lysosome-like vacuole is the major degradative organelle and a storage site for metabolites, amino acids and ions essential for cellular fitness during times of nutrient excess and scarcity^1^. Macromolecular degradation and metabolite storage in the lysosome/vacuole requires the maintenance of an acidic lumen relative to the cytosol, which is largely achieved by the highly conserved, ATP-dependent, proton-pumping vacuolar ATPase (v-ATPase)^1^. Regulation of v-ATPase activity functionalizes the vacuole to make proliferative decisions for the cell. Through a reciprocal relationship with the master growth regulator TORC1, the vacuole integrates diverse metabolic cues such as glycolytic flux and nitrogen availability to enact anabolic or catabolic cellular pathways^2^. Thus, regulation of the v-ATPase, vacuolar pH and TORC1 signaling are functionally intertwined to read out various aspects of cellular metabolism to make grow/no-grow decisions.

An important function of the vacuole in regulating cellular proliferative decisions comes about by its ability to store amino acids. Notably, the degree of vacuolar amino acid compartmentalization is very specific to particular classes of amino acids. In yeast, approximately 90% of intracellular basic amino acids and 10% of acidic amino acids are stored in the vacuole, whereas all other classes of amino acids are only moderately enriched^3^.

The functional importance of discrete amino acid compartmentalization during cell growth in nutrient-replete conditions is not well understood; however, one consequence of dysregulated compartmentalization has recently been shown. As yeast cells age, vacuolar pH increases and cytoplasmic cysteine levels increase, which impacts mitochondrial function by altering iron–sulfur cluster biogenesis and the organelle’s ability to carry out critical activities^4,5^. This insight into the importance of vacuolar pH-dependent cysteine compartmentalization on mitochondrial function highlights the interconnectivity of organellar homeostasis during aging but raises the question whether there is any role for the compartmentalization of different classes of amino acids in young exponentially growing cells.

While many details regarding the regulation of vacuolar acidity and metabolite storage have been discovered under conditions of cell stressors such as starvation, osmotic shock or aging, the dynamics of vacuolar pH homeostasis under constant growth conditions has not been explored due to a paucity of molecular tools. Specifically, it has not been possible to observe vacuolar pH dynamics in single yeast cells over long periods of time with high temporal resolution and sensitivity.

Here, we explored the interconnectivity of vacuolar pH regulation, cellular amino acid homeostasis and the cell cycle in yeast. We developed a new fluorescent reporter optimized for the lower pH of the vacuole lumen and used it to discover a previously unappreciated cell cycle-linked regulation of vacuolar pH in cells growing in the presence of particular amino acids. We identified several highly conserved molecular pathways regulating these dynamics and provide evidence for the cycling of the vacuolar lipid phosphatidylinositol 3,5-bisphosphate (PtdIns(3,5)P_2_), known to be important in coordinating the activity of TORC1 and the v-ATPase. Furthermore, we showed that these pH changes are controlling amino acid accumulation in and release from, the vacuole. Blocking dynamic vacuolar pH alkalinization in nutrient-replete conditions leads to an upregulation of cytoplasmic arginine biosynthetic genes and the concordant downregulation of mitochondrial oxidative phosphorylation and ribosome genes. Phenotypically, these lead to the dysregulated coordination of the size at which daughter cells are produced and the time they spend in G_1_ before committing to a new round of DNA synthesis.

These data provide evidence for a previously unanticipated cell-cycle-linked homeostatic control of cytoplasmic amino acid levels and present a new model for the regulation of vacuolar pH and amino acid compartmentalization during cell growth.

## Results

### Vacuolar pH is oscillatory in single cells over time

**Fig. 1:**
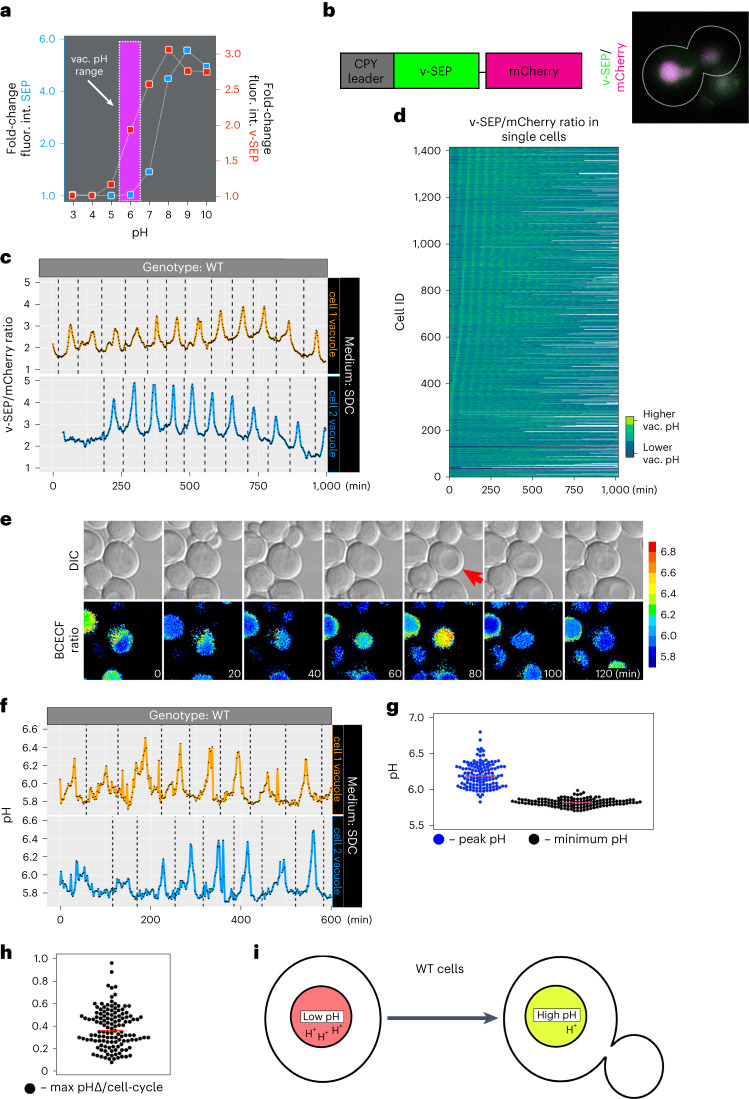
Vacuolar pH is dynamic and oscillates during cell growth. **a**, Fluorescence intensity fold-change of SEP (blue squares) and v-SEP (red squares) from cells permeabilized in situ with digitonin and exposed to solutions of different pH. Fluorescence was measured with a spectrophotometer. The expected vacuolar pH range is boxed in magenta. **b**, A schematic of the ratiometric pH-sensitive reporter composed of the acid sensitive v-SEP fluorophore and the acid insensitive mCherry fluorophore targeted to the vacuole by the N-terminal fusion of the first 50 amino acids of carboxypeptidase Y (CPY leader). The micrograph shows both fluorophores localized to the vacuole in a single cell. **c**, Time-series data show the ratio of vacuolar v-SEP intensity to mCherry intensity over time for two representative cells grown in medium containing glucose and amino acids (SDC) and imaged every 2 min for 1,000 min in microfluidics chambers (CellASIC). Dashed lines indicate the timing of bud emergence. **d**, The 1,417 single cell ratiometric fluorescence traces of v-SEP/mCherry in cells grown in custom-built microfluidics devices in the presence of SDC. Fluorescence data are aligned to the first peak of vacuolar pH alkalinization. **e**, Single frames of timelapse data show brightfield and the ratiometric signal of vacuolar accumulated BCECF. Absolute pH values are calculated from in situ generated standard curves. A red arrow highlights the peak of a vacuolar alkalinization event and the change in refractive index in the brightfield panel. **f**, Absolute pH changes calculated from ratiometric BCECF imaging plotted over time for two representative cells. **g**, Peak pH and minimum pH observed over 129 cell cycle events from 27 different cells. Average values are denoted by horizontal red bars. **h**, The difference between peak and minimum pH values within 129 discrete cell cycle events from 27 cells. Average value is denoted by a horizontal red bar. **i**, A schematic model shows vacuolar pH changes as wild-type (WT) cells progress through the cell cycle. In unbudded cells (left), vacuolar pH is more acidic and as cells grow (right), the organelle becomes less acidic. Source data

The lack of robust tools to monitor pH changes in acidic organelles prompted us to create a new reporter. For this, we mutated the pH-sensitive fluorescent protein, super-ecliptic pHluorin (SEP)^6^, shifting its fluorescence pKa to be more sensitive to changes in acidic environments. We introduced mutations A227D/D147S based on their effect on the pH dependence of the fluorescent domain of ArcLight^7^ and named the resulting fluorophore, v-SEP (Fig. 1a). To create a ratiometric vacuolar-localized reporter, v-SEP was fused to the pH-insensitive red fluorophore mCherry and directed to the lumen of the vacuole using the first 50 amino acids of the vacuolar hydrolase carboxypeptidase Y (CPY) (Fig. 1b).

We used microfluidics devices to immobilize fully prototrophic cells and imaged v-SEP and mCherry every 2 min for 1,000 min (approximately ten cell divisions) during growth in a constant, defined extracellular environment (growth medium with essential nutrients, amino acids and glucose (SDC)).

**Extended Data Fig. 1:**
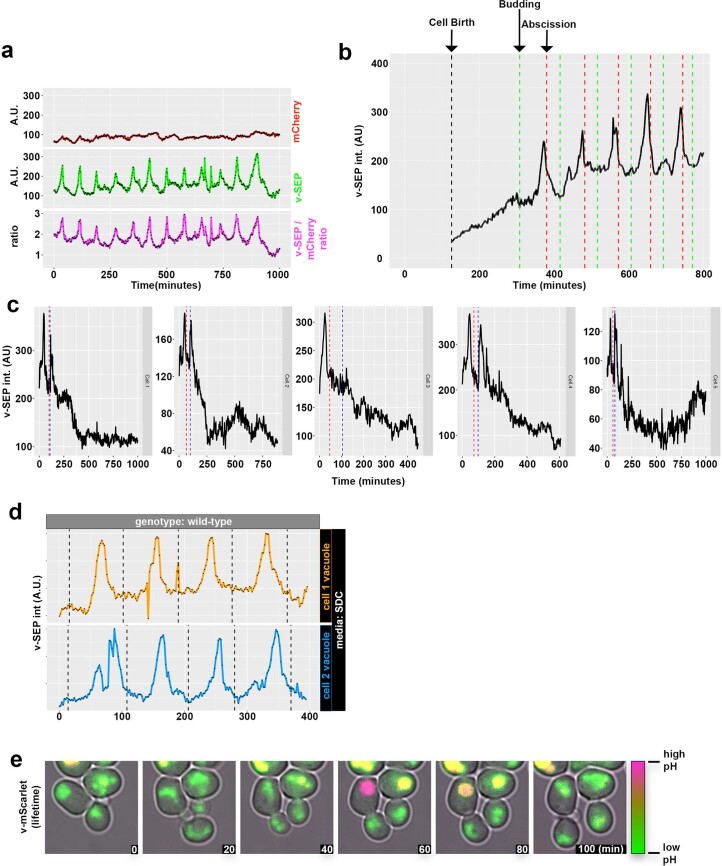
Supporting data for Figure 1. **(a)** Individual mCherry and v-SEP intensities and the ratio of the two signals imaged every 2 minutes for 1000 minutes. **(b)** Cells expressing v-SEP and Psr1-mRuby2 imaged every 2 minutes while growing in SDC. Fluorescence intensity changes of v-SEP from one cell is shown in black while the dashed vertical bar indicates the time at which the cell was born. Vertical green and red dashed lines indicate the timing of each daughter cell initiation and separation respectively. **(c)** *bar1∆* cells expressing v-SEP imaged every 2 minutes while growing in SDC with 200 nM α-factor. Cells undergo one cell division (cell separation indicated by dashed red line) before arresting (indicated by dashed blue line). **(d)** Cells expressing v-SEP were grown in batch culture in SDC and allowed to settle onto a coverslip and imaged every 2 minutes. v-SEP intensity changes over time for two cells is shown. **(e)** Cells expressing v-mScarlet were grown in SDC and fluorescence lifetimes were captured as described in the Material and Methods. Fluorescence lifetimes were pseudo-colored and overlaid on brightfield images with longer lifetimes represented in magenta and shorter lifetimes in green, corresponding to higher and lower pH respectively. Source data

Our analysis revealed a notable dynamic aspect of vacuolar pH regulation. We observed regular increases and decreases in the ratio of v-SEP to mCherry fluorescence (Fig. 1c and Supplementary Video 1) which, when correlated with the timing of bud emergence (Fig. 1c; dashed vertical lines), happened once per cell cycle late in mitosis. mCherry fluorescence was minimally changed over the imaging period (Extended Data Fig. 1a) and identical results were seen imaging v-SEP alone (Fig. 1 and Supplementary Video 2). To more precisely time the alkalinization and acidification events within the cell cycle, we labeled the plasma membrane with mRuby2 fused to the first 28 residues of the palmitoylated plasma membrane-associated phosphatase, Psr1 and imaged v-SEP and Psr1-mRuby2 in growing cells. Using Psr1-mRuby2 allowed us to precisely time new daughter initiation (Extended Data Fig. 1b; dashed green vertical lines) and cell separation (Extended Data Fig. 1b; dashed red vertical lines) relative to v-SEP intensity changes (Extended Data Fig. 1b; black line). We quantified the timing across 14 cell cycle events from bud emergence to peak vacuolar alkalinization (59.4 ± 4.6 min) and the timing from peak vacuolar alkalinization to cell separation (5.7 ± 1.4 min). To test whether cell cycle progression is important for oscillatory vacuolar pH dynamics, we arrested *MATa* cells in G_1_ with ɑ-factor^8^ and observed no oscillations after cell cycle arrest (Extended Data Fig. 1c; red dashed line shows cell separation, blue dashed line shows cell cycle arrest). This analysis shows that oscillatory vacuolar pH dynamics require cell cycle progression and that vacuolar alkalinization peaks just before cell separation in late anaphase/telophase.

To ascertain whether the microfluidics device impacted vacuolar pH dynamics, cells were grown in batch culture in SDC medium and allowed to settle directly on coverslips. v-SEP was imaged for ~400 min or approximately four cell divisions. We observed identical dynamics of the v-SEP reporter under these conditions (Extended Data Fig. 1d and Supplementary Video 3), suggesting that the microfluidics device did not cause the oscillatory behavior. We also determined whether cell crowding and local nutrient deprivation in the microfluidics device might influence vacuolar pH dynamics. To this end we used a custom-built microfluidics platform^9^ wherein single cells were trapped in individual cell catchers and daughter cells were washed away under constant medium flow. Ratiometric fluorescence data (v-SEP/mCherry) acquired over ~1,000 min was aligned to the first identified vacuolar alkalinization event in 1,417 single cells (Fig. 1d). We observed robust oscillations under this experimental regime reinforcing the conclusion that neither the microfluidics device nor cell crowding/nutrient deprivation are influencing vacuolar pH dynamics in our experiments.

We next examined vacuolar pH dynamics with two additional, independent approaches. First, we applied temporally resolved fluorescence lifetime imaging microscopy (FLIM) to measure the pH-dependent changes in the fluorescence lifetime of vacuole-targeted mScarlet (v-mScarlet). The fluorescence lifetime of mScarlet is sensitive to its local pH, but insensitive to local ion composition and fluorophore concentration^10^. Fluorescence lifetime changes showed very similar alkalinization and reacidification dynamics in the vacuole as was seen with the v-SEP reporter (Extended Data Fig. 1e and Supplementary Video 4).

Second, we used the vacuole-sequestered pH-sensitive, ratiometric dye, BCECF^4,5^. Cells were grown in SDC, treated with BCECF in the same medium and then imaged over time in the microfluidics device using pH-sensitive excitation, pH-insensitive excitation and brightfield (Fig. 1e and Supplementary Video 5). This analysis also revealed oscillatory pH dynamics (Fig. 1f). Using a calibration curve, we quantified the pH changes across 129 cell cycle events for 27 cells. The lowest pH across cell cycles was relatively constant, 5.82 (95% confidence interval (CI) 5.81–5.83) (Fig. 1g; black points), while the peak alkalinization events on average were more variable and averaged pH 6.20 (95% CI 6.18–6.23) (Fig. 1g; blue points). The average pH change per cell cycle was 0.38 pH units (95% CI, 0.36–0.41) (Fig. 1h).

Taken together, these data suggest that v-SEP accurately reports changes in vacuolar pH. Thus, vacuoles in cells growing in a constant defined growth environment containing glucose and amino acids, either in batch culture or in microfluidics devices, undergo regular oscillatory alkalinization and acidification events once per cell cycle (Fig. 1i).

### Quantitative analysis of vacuolar pH oscillations

**Fig. 2:**
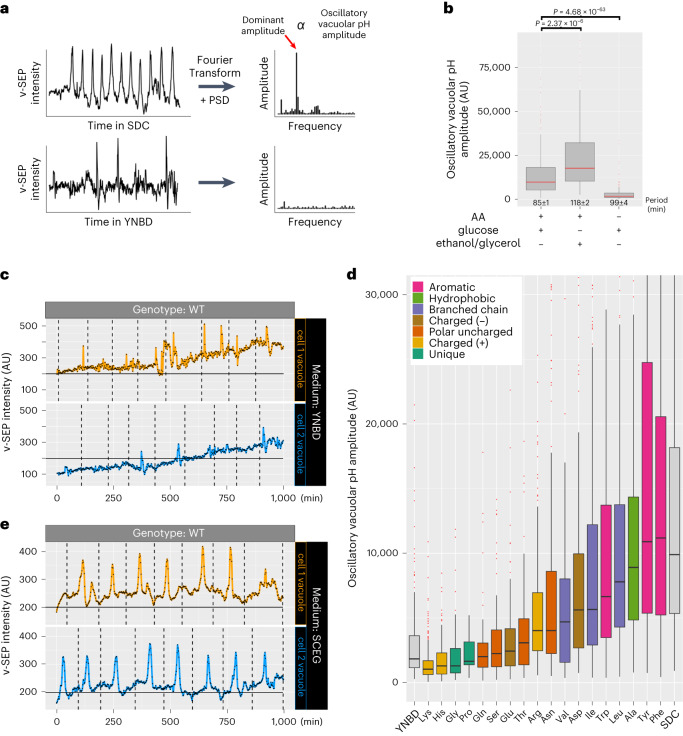
Vacuolar pH dynamics are sensitive to extracellular amino acids and alanine, branched-chain and aromatic amino acids are sufficient to induce oscillations. **a**, A schematic of the PSD pipeline to transform v-SEP intensity time-series data from cells grown in SDC (top) or YNBD (bottom) to amplitude versus frequency plots. Dominant amplitude is proportional to the magnitude of oscillatory pH changes over the imaging period (oscillatory vacuolar pH amplitude). **b**, Oscillatory vacuolar pH amplitude was obtained using the PSD pipeline for 339 cells grown in SDC (+amino acids (AA) +glucose), 133 cells grown in SCEG (+AA, +ethanol/glycerol) and 321 cells grown in YNBD (−AA, +glucose). Median vacuolar pH amplitude is displayed as box-and-whisker plots with whiskers calculated as 1.5 × the interquartile ranges, median values indicated by the horizontal red bar and *P* values are calculated by a two-sided Dunn’s test with Holm adjustment. Numbers inset below bar graphs are the median period of vacuolar pH oscillations. **c**, Cells grown in YNBD imaged every 2 min for 1,000 min in microfluidics chambers (CellASIC). v-SEP intensity over time is shown for two representative cells. Vertical dashed lines indicate the timing of bud emergence. **d**, Oscillatory vacuolar pH amplitude from cells grown in the presence of individual amino acid addbacks. Median vacuolar pH amplitude is displayed as box-and-whisker plots with whiskers calculated as 1.5 × the interquartile ranges, median values indicated by the horizontal black bar. Note, the *y* axis is fixed at 30,000 AU. Cell number used in the analysis and periodicity of vacuolar pH oscillations in minutes for each condition: Lys (*n* = 255, 111 ± 3 min), His (*n* = 132, 110 ± 5 min), Gly (*n* = 92, 104 ± 10 min), Pro (*n* = 26, 82 ± 18 min), Gln (*n* = 64, 96 ± 11 min), Ser (*n* = 60, 95 ± 8 min), Glu (*n* = 90, 87 ± 10 min), Thr (*n* = 54, 91 ± 13 min), Arg (*n* = 280, 110 ± 3 min), Val (*n* = 56, 106 ± 10 min), Asn (*n* = 163, 105 ± 3 min), Asp (*n* = 102, 100 ± 10 min), Ile (*n* = 420, 98 ± 2 min), Trp (*n* = 121, 112 ± 4 min), Leu (*n* = 356, 104 ± 2 min), Ala (*n* = 157, 97 ± 3 min), Phe (*n* = 429, 90 ± 2 min) and Tyr (*n* = 334, 101 ± 2 min). **e**, Cells grown in SCEG imaged every 2 min for 1,000 min in microfluidics chambers (CellASIC). v-SEP intensity over time is shown for two representative cells. Dashed lines indicate the timing of bud emergence. See Source Data for additional curves. Source data

Vacuole pH is responsive to changes in extracellular environment; therefore, to compare vacuolar pH oscillations in cell populations growing in different growth environments and from different genetic backgrounds, we quantified the period (the frequency of alkalinization) and the magnitude of vacuolar pH changes in individual cells progressing through repeated cell cycles. We applied automated feature segmentation and tracking to extract the temporally resolved fluorescence intensity changes of v-SEP in individual vacuoles as they transitioned through at least five cell divisions. The trajectories were Fourier transformed to obtain the power spectral density (PSD) of the time-series data from wild-type cells grown in SDC (Fig. 2a; top trace). In this situation, power is proportional to fluorescence intensity (oscillatory vacuolar pH amplitude) over a particular time domain. The frequency with the greatest power indicates the dominant pH oscillation frequency. We calculated the median of the amplitudes at the dominant frequency for ~300 cells growing in the microfluidics devices. These cells had a vacuolar pH amplitude of 9,900 arbitrary units (AU) (95% CI 9,100–11,000) and a median periodicity of 84.9 min (95% CI 84.1–85.9, Wilcoxon signed-rank test) (Fig. 2b; +AA +glucose). These data are consistent with oscillations occurring once every cell cycle under these growth conditions.

**Extended Data Fig. 2:**
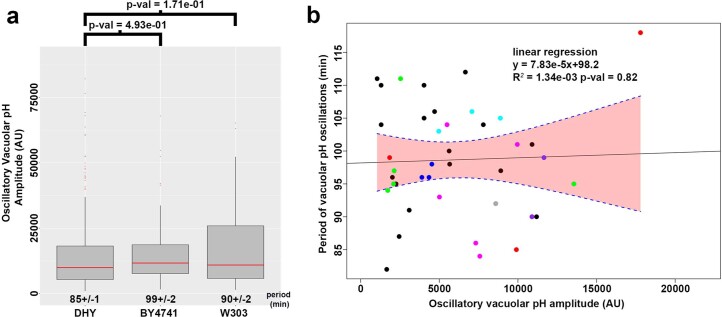
Supporting data for Figure 2. **(a)** Oscillatory vacuolar pH amplitude of DHY (n = 301), BY4741 (n = 99) and W303 (n = 90) wild-type cells grown in media containing glucose and amino acids (SDC). Median vacuolar pH amplitude is displayed as box-and-whisker plots with whiskers calculated as 1.5 * the interquartile ranges, median values indicated by the horizontal red bar and p values are calculated by a two-sided Dunn’s test with Holm adjustment. Numbers inset below bar graphs are the median period of vacuolar pH oscillations. **(b)** Vacuolar pH period and magnitude is plotted for the experiments shown in Fig. 2b (red), 2d (black), 3a (blue), 3d (green), 3e (magenta), 3 f (cyan), Extended Data Fig. 2a (grey) and Extended Data Fig. 3b (violet). Solid horizontal black line is the regression line and the shaded red area is the 95% confidence interval around the regression line. Source data

To determine whether the oscillatory pH dynamics were unique to the strain examined here, the analysis pipeline was used to examine v-SEP fluorescence dynamics in two different, but commonly used, laboratory strains, W303 and BY4741. We found that both had robust cell cycle-linked oscillatory pH dynamics of similar amplitude and period (Extended Data Fig. 2a).

### Vacuolar pH dynamics depend on extracellular amino acids

The vacuole is a central integrator of extracellular nutritional availability and a major site of amino acid storage^1^. This led us to examine whether extracellular amino acids impacted oscillatory vacuolar pH dynamics. To this end, wild-type cells were cultured in medium that contained glucose, salts and essential nutrients, but lacked amino acids (YNBD). Vacuolar pH dynamics were then examined with the v-SEP reporter. In contrast to cells grown in SDC, which provides a full complement of exogenously supplied amino acids (Fig. 1c), cells grown in YNBD did not undergo clear and sustained oscillatory vacuolar alkalinization and acidification cycles (Fig. 2c and Supplementary Video 6). Instead, we observed a slow progressive increase in v-SEP intensity as cells went through repeated cell divisions; it is unknown why this rise occurs. In addition, vacuoles underwent stochastic pulses of alkalinization and reacidification that were not apparently linked to any stage of the cell cycle. The absence of regular oscillatory dynamics was supported by PSD analysis (Fig. 2a; bottom trace), which showed that any oscillatory behavior of v-SEP was marginally above background for cell populations grown in YNBD (Fig. 2b; −AA +glucose). Moreover, these stochastic alkalinization and reacidification events were notably quicker than seen in SDC. The median half-maximal peak width of time spent in an alkaline state averaged 2 min in YNBD (limit of time resolution for the experiments) versus 11 min in SDC.

The clear difference in the amplitude of oscillatory vacuolar pH dynamics between cells grown in the presence or absence of amino acids prompted us to ask whether any individual, or classes of, amino acids were contributing to these dynamics. To this end, cells were grown in YNBD with single amino acids added at concentrations found in SDC and the amplitude of the oscillatory dynamics of the cell population using PSD analysis was assessed. As reported previously, cysteine alone caused cells to grow slowly^11,12^ and in our hands freshly prepared methionine alone also caused growth defects so they were omitted from further analysis. For the remaining amino acids, we observed a graded response in the oscillatory vacuolar pH amplitude. Some, such as lysine, glycine, histidine and proline were barely able to induce vacuolar pH oscillations (Fig. 2). By contrast, the aromatic amino acids tyrosine and phenylalanine, the branched-chain amino acid leucine and alanine tended to induce robust vacuolar pH oscillations Fig. 2), whereas all other amino acids produced intermediate responses (Fig. 2d).

We noticed during our amino acid supplementation experiments that some amino acids, such as tryptophan and glutamine, caused cells to have longer and shorter periods of vacuolar pH oscillation respectively, raising the possibility that growth rate might influence the magnitude of vacuolar pH changes. To test whether cell growth rate and changes in vacuolar pH oscillation period might be affecting the magnitude of the oscillations, we plotted the period of vacuolar pH oscillations relative to their magnitude for all growth conditions and mutants tested in this manuscript (described below) and found no correlation between the two parameters (Extended Data Fig. 2b), suggesting that growth rate does not influence the magnitude of vacuolar pH changes.

Glycolytic and respiratory carbon sources are well documented regulators of vacuolar pH^13^. Therefore, we examined whether carbon source affects vacuolar pH dynamics during the cell cycle. Cells were grown in medium containing all amino acids and ethanol/glycerol (SCEG), a respiratory carbon source. There was a ~1.8-fold increase in the oscillatory vacuolar pH amplitude, at the population level (18,000 AU, 95% CI 14,000–22,000, *n* = 133 cells), relative to cells grown in glucose (Fig. 2b; compare +AA +ethanol/glycerol to +AA +glucose; and Supplementary Video 7). Individual v-SEP intensity traces showed readily identifiable cell cycle-linked oscillations with increased amplitude and increased basal intensity relative to cells grown in the presence of glucose (Fig. 2e; compare representative curves in Fig. 1 (v-SEP.SDC) with Fig. 2 (v-SEP.SCEG)). This is consistent with data suggesting that the availability of glycolytic carbon sources is a major regulator of vacuolar pH^13^.

Taken together, these data suggest that the amplitude of vacuolar pH oscillations are modulated by aromatic and branched-chain amino acid availability and the fermentative or respiratory capacity of the supplied carbon source.

### Vacuolar pH dynamics require multiple cell pathways

**Fig. 3:**
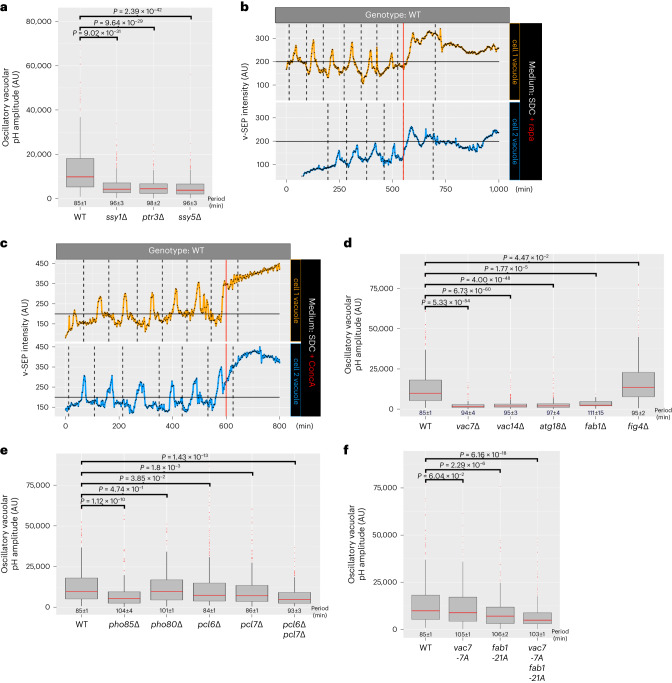
Vacuolar pH oscillations require the SPS response, TORC1 signaling, v-ATPase activity, oscillatory production of PtdIns(3,5)P_2_ by the Fab1 complex and the nutrient-responsive CDK, Pho85. **a**, Oscillatory vacuolar pH amplitude of wild-type cells and cells lacking the SPS components, Ptr3 (288 cells), Ssy1 (447 cells) and Ssy5 (399 cells), grown in SDC. Median vacuolar pH amplitude is displayed as box-and-whisker plots with whiskers calculated as 1.5 × the interquartile ranges, median values indicated by the horizontal red bar and *P* values are calculated by a two-sided Dunn’s test with Holm adjustment. Numbers inset below bar graphs are the median period of vacuolar pH oscillations. **b**, Cells grown in SDC imaged every 2 min for 1,000 min in microfluidics chambers (CellASIC) and treated in the device with rapamycin (vertical red bar). v-SEP intensity over time is shown for two representative cells. **c**, Cells grown in SDC imaged every 2 min for 1,000 min in microfluidics chambers (CellASIC) and treated in the device with ConcA (vertical red bar). v-SEP intensity over time is shown for two representative cells. Dashed lines indicate the timing of bud emergence. **d**, Oscillatory vacuolar pH amplitude of WT cells and cells lacking the Fab1 components, Vac7 (177 cells), Vac14 (287 cells), Atg18 (200 cells), Fab1 (21 cells) and Fig4 (206 cells), grown in SDC. Median vacuolar pH amplitude is displayed as box-and-whisker plots with whiskers calculated as 1.5 × the interquartile ranges, median values indicated by the horizontal red bar and *P* values are calculated by a two-sided Dunn’s test with Holm adjustment. Numbers inset below bar graphs are the median period of vacuolar pH oscillations. **e**, Oscillatory vacuolar pH amplitude of wild-type cells and cells lacking the CDK5 homolog Pho85 (201 cells) and associated cyclins Pho80 (240 cells), Pcl6 (315 cells), Pcl7 (277 cells) and the Pcl6 and Pcl7 double mutant (206 cells). Cells were grown in SDC. Median vacuolar pH amplitude is displayed as box-and-whisker plots with whiskers calculated as 1.5 × the interquartile ranges, median values indicated by the horizontal red bar and *P* values are calculated by a two-sided Dunn’s test with Holm adjustment. Numbers inset below bar graphs are the median period of vacuolar pH oscillations. **f**, Oscillatory vacuolar pH amplitude of WT cells and cells lacking seven putative Pho85 phospho-sites in Vac7 (*vac7-7A*, 228 cells) and 21 putative phospho-sites in Fab1 (*fab1-21A*, 168 cells) and the double mutant (*vac7-7A fab1-21A*, 257 cells). Cells were grown in SDC. Median vacuolar pH amplitude is displayed as box-and-whisker plots with whiskers calculated as 1.5 × the interquartile ranges, median values indicated by the horizontal red bar and *P* values are calculated by a two-sided Dunn’s test with Holm adjustment. Numbers inset below bar graphs are the median period of vacuolar pH oscillations. See Source Data for additional curves. Source data

**Extended Data Fig. 3:**
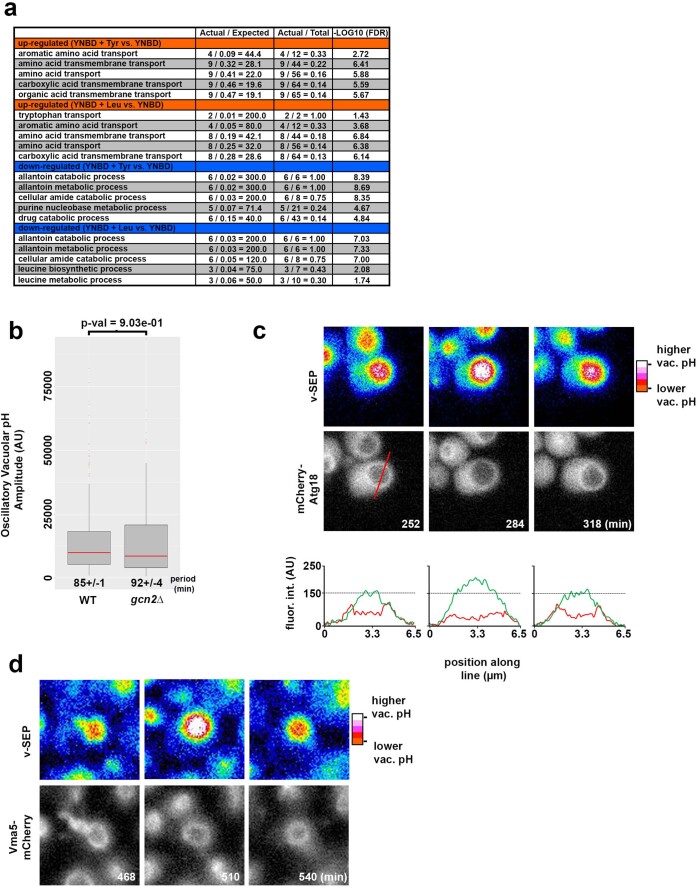
Supporting data for Figure 3. **(a)** Table of upregulated and downregulated GO terms comparing wild-type cells growing in YNBD vs. YNBD + Tyr or YNBD + Leu. **(b)** Oscillatory vacuolar pH amplitude of *gcn2∆* and wild-type cells grown in media containing glucose and amino acids (SDC). Median vacuolar pH amplitude is displayed as box-and-whisker plots with whiskers calculated as 1.5 * the interquartile ranges, median values indicated by the horizontal red bar and p values are calculated by a two-sided Dunn’s test with Holm adjustment. Numbers inset below bar graphs are the median period of vacuolar pH oscillations. **(c)** Single frames from timelapse imaging of mCherry-Atg18 and v-SEP during one round of vacuolar alkalinization (same as Fig. 4a). The lower plots show mCherry-Atg18 intensity (red trace) and v-SEP intensity (green trace) along the red line indicated in the mCherry-Atg18 image. A reference horizontal dashed line is set at 150 AU for all plots. **(d)** Single frames from timelapse imaging of Vma5-mCherry and v-SEP during one round of vacuolar alkalinization. Source data

To identify cellular pathways that might be sensing and responding to increased extracellular branched-chain and aromatic amino acids, we analyzed the transcriptional signature of cells grown in the presence of tyrosine or leucine by RNA-seq. As previously demonstrated^14^, cells responded to extracellular aromatic and branched-chain amino acids by upregulating the expression of amino acid permeases through the action of the SPS response (Extended Data Fig. 3a). The SPS response is driven by a sensor module at the plasma membrane composed of Ssy1, Ptr3 and Ssy5, which activates two transcription factors, Stp1 and Stp2^15^. The SPS response induces the synthesis of ~ten amino acid permeases that drive amino acid import into the cell^16^. To test the role of the SPS response and amino acid import in regulating vacuolar pH, we quantified the oscillatory vacuolar pH amplitude in cells lacking SPS-response components grown in SDC (Fig. 3a). In each mutant, we observed a >twofold reduction in the oscillatory vacuolar pH amplitude relative to wild-type cells (Fig. 3a). These data suggest that the increase in intracellular amino acids that results from the SPS response is important to stimulate the oscillatory pH dynamics in the vacuole.

To identify a molecular link between changes in cytoplasmic branched-chain and aromatic amino acid availability and oscillatory vacuolar pH amplitude, we tested the contribution to the induction of vacuolar pH oscillations by the two major cytoplasmic amino acid-sensing pathways: general amino acid control and TORC1^15,17^. While deletion of the eIF2-kinase, *GCN2*, did not affect oscillatory vacuole pH dynamics (Extended Data Fig. 3b), pharmacological inhibition of TORC1 signaling by rapamycin treatment of cells had a strong effect (Fig. 3b; timing of rapamycin addition marked by red vertical line; Supplementary Video 8).

TORC1 signaling integrates cellular nutritional status with vacuole/lysosome metabolite storage through v-ATPase function in yeast and mammalian cells^2^. Additionally, the v-ATPase is the major regulator of vacuolar pH. Therefore, we tested the contribution of the v-ATPase to oscillatory vacuolar pH dynamics by genetically deleting the vacuole-specific V0 subunit of the v-ATPase, Vph1. We found, as previously reported, that cells lacking Vph1 have highly fragmented vacuoles^18^; these highly fragmented vacuoles in *vph1∆* cells were exceptionally mobile and too difficult to accurately track and quantify over the course of many hours. To circumvent this challenge, we tested for a direct role for the v-ATPase by treating cells with the pharmacological v-ATPase inhibitor, concanamycin A (ConcA). After switching to growth medium containing the v-ATPase inhibitor (Fig. 3c, timing ConcA addition marked by red vertical line, and Supplementary Video 9), the fluorescence intensity of v-SEP progressively increased, indicating vacuolar alkalinization and loss of oscillatory pH dynamics. Notably, the increase in fluorescence of the v-SEP reporter preceded the fragmentation seen in *vph1∆* cells which permitted accurate tracking and quantification of v-SEP fluorescence changes. Taken together, these data suggest that the v-ATPase is a major facilitator of cell cycle-linked vacuole pH oscillations.

**Fig. 4:**
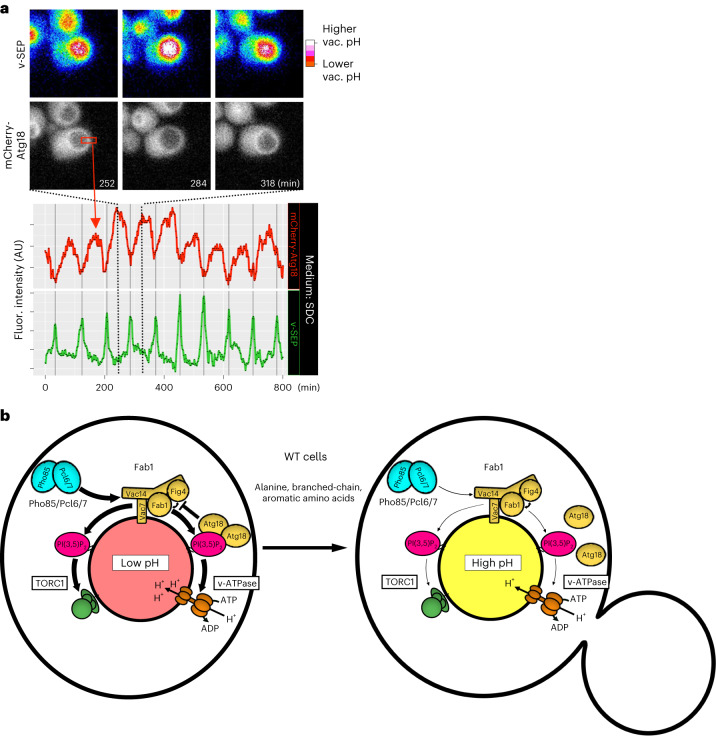
Atg18 undergoes rounds of recruitment and displacement from the vacuolar surface during vacuolar pH dynamics. **a**, Single frames from timelapse imaging of mCherry-Atg18 and v-SEP during one round of vacuolar alkalinization highlighted by dashed lines in the time-series plot below. The lower time-series plot shows mCherry-Atg18 intensity at the vacuolar surface and lumenal v-SEP intensity over multiple cell division events. Peak v-SEP signals are highlighted by a solid gray line. **b**, A model shows the molecular players involved in regulation of vacuolar pH as WT cells grow in the presence of alanine, branched-chain and aromatic amino acids. pH regulation requires Pho85/Pcl6/Pcl7-dependent stimulation of the Fab1 (PIKfyve in mammals) complex to produce PtdIns(3,5)P_2_ (PI(3,5)P_2_), which recruits Atg18 and reciprocally activates TORC1 and the v-ATPase. During growth, PtdIns(3,5)P_2_ levels at the vacuole drop, which may in turn downregulates both TORC1 and v-ATPase leading to an increase in vacuolar pH. See Source Data for additional curves. Source data

To explore the inputs that may couple regulation of the v-ATPase and TORC1 activity, we examined the role of the vacuole-specific lipid PtdIns(3,5)P_2_ in contributing to oscillatory vacuolar pH dynamics. PtdIns(3,5)P_2_ is a stress-responsive, minority phosphoinositol lipid of the vacuole. It is produced by the Fab1 (PIKfyve in mammals) kinase complex, which contains Fab1, Vac7, Vac14 and is negatively regulated by Fig4 and Atg18^19^. PtdIns(3,5)P_2_ interacts with and stabilizes the v-ATPase holoenzyme in osmotically stressed cells and plays a role in regulating TORC1 signaling during nutritional stress^19^. The Fab1 complex is also reciprocally regulated by TORC1^20^, making PtdIns(3,5)P_2_ production a potentially important regulatory crossroad to coordinate vacuolar pH dynamics and amino acid sensing. While it has been suggested that the Fab1 complex does not affect vacuolar pH in unstressed rapidly growing cells^21^, the dynamics of pH regulation has not been examined in Fab1 mutants at the single cell level. Genetic deletion of Vac7, Vac14, Atg18 and Fab1 had a strong effect on the amplitude of oscillatory pH dynamics in the presence of extracellular amino acids relative to wild-type cells (Fig. 3d). Of note, deletion of Fig4, a phosphatase proposed to act as an important regulator of the complex^22^, had a slightly higher oscillatory vacuolar pH amplitude relative to wild-type cells in SDC (Fig. 3d). These data suggest that PtdIns(3,5)P_2_ production is essential for modulating oscillatory vacuolar pH dynamics and that the regulation of the Fab1 complex by Fig4 is not strictly required but plays a more nuanced regulatory role.

PtdIns(3,5)P_2_ production during osmotic stress is linked to cyclin-dependent kinase (CDK) signaling through the activity of the nutrient-responsive CDKcyclin pair Pho85–Pho80^19^. Pho85–Pho80 directly phosphorylates the Fab1 complex and positively regulates its activity^19^. To test whether CDK signaling influences oscillatory vacuolar pH dynamics, we quantified the amplitude of pH oscillations in cells lacking Pho85 and Pho80. The *pho85∆* cells had strongly reduced oscillations, whereas oscillations in *pho80∆* cells were not substantially reduced (Fig. 3e) relative to wild-type cells (Fig. 3e), suggesting that, similar to the Fig4 example above for the Fab1 complex, aspects of PtdIns(3,5)P_2_ regulation during unstressed cell growth are different than during osmotic stress. Pho85 has ten cyclin partners (Pho80, Clg1, Pcl1, Pcl2, Pcl5, Pcl6, Pcl7, Pcl8, Pcl9 and Pcl10) that are composed of paralogous pairs of cyclins with redundant functions^23^. We found Pcl6 and Pcl7 each contributed to vacuolar pH oscillations (Fig. 3e). Deletion of both Pcl6 and Pcl7 (Fig. 3e) further reduced the amplitude of oscillatory vacuolar pH dynamics to that seen in *pho85∆* cells.

Pho85 is proposed to phosphorylate Fab1 and Vac7 to regulate their activity^19^. To test the importance of Pho85-dependant phosphorylation of Fab1 and Vac7 for vacuolar pH dynamics, we quantified the amplitude of pH oscillations in previously described mutants of Fab1 lacking 21 putative Pho85 phospho-sites (*fab1-21A*) and Vac7 lacking 7 putative Pho85 phospho-sites (*vac7-7A*)^19^. *Vac7-7A* mutant cells had slightly reduced vacuolar pH oscillations, whereas *fab1-21A* mutant cells had a stronger deficit (Fig. 3f). The double mutant further reduced oscillatory vacuolar pH amplitude to that seen in the *pcl6∆pcl7∆* and *pho85∆* cells (Fig. 3f).

Taken together these data suggest Pcl6 and Pcl7 are two cyclins which act redundantly through the Pho85 kinase to regulate cell cycle-linked oscillatory vacuolar pH dynamics, most likely by phosphorylation of Fab1 and Vac7.

### PtdIns(3,5)P_2_ dynamics drive vacuolar pH regulation

The data thus far suggest a model where Pho85/Pcl6/Pcl7-dependent changes in Fab1 activity alter PtdIns(3,5)P_2_ levels to affect v-ATPase activity and TORC1 function. However, to date, there is no indication that PtdIns(3,5)P_2_ levels in the vacuole are changing during cell growth to enact such regulatory control during the cell cycle. Measuring PtdIns(3,5)P_2_ in unstressed cells is challenging due to its low abundance relative to the total phosphoinositol pool. Additionally, there is evidence that PtdIns(3,5)P_2_ production is differentially regulated between signaling endosomes and the vacuole^20^, which makes techniques that measure total cellular PtdIns(3,5)P_2_ levels inappropriate to capture the dynamics of this lipid specifically at the vacuole. Atg18 is an important regulatory component of PtdIns(3,5)P_2_ generation and is proposed to be a PtdIns(3,5)P_2_ sensing protein. It is also a negative regulator of the Fab1 complex. To explore the accumulation of PtdIns(3,5)P_2_ in the vacuole, we monitored the dynamics of Atg18 along with the v-SEP reporter. N-terminally tagged mCherry-Atg18 showed clear vacuolar limiting membrane localization in cells with acidic vacuoles (Fig. 4a, top left, and Supplementary Video 10); however, as vacuoles became less acidic, mCherry-Atg18 was displaced from the membrane and then re-assembled on the vacuolar surface as acidity was re-established (Fig. 4a, top middle and right, and Supplementary Video 10). Quantification of mCherry-Atg18 at the vacuolar surface relative to v-SEP fluorescence shows that Atg18 undergoes rounds of recruitment and displacement from the vacuolar surface which are strictly tied to rounds of acidification and alkalinization, respectively (Fig. 4a, bottom plot, and Extended Data Fig. 3c). v-ATPase activity and assembly are influenced by PtdIns(3,5)P_2_. To test whether cycling PtdIns(3,5)P_2_ levels in unstressed cells affects v-ATPase assembly state, we tagged a subunit of the peripheral V_1_ domain (Vma5) and examined its localization over one cell cycle together with the v-SEP reporter. We found no evidence that the V_1_ domain of the v-ATPase is regulated at the level of assembly at the vacuolar surface (Extended Data Fig. 3d). Together, these data suggest that PtdIns(3,5)P_2_ levels are cycling throughout the cell cycle and provides evidence that PtdIns(3,5)P_2_-dependent regulation of the v-ATPase is acting beyond the level of assembly status/osmotic stress and may play a role regulating vacuolar pH during vegetative growth in unstressed cells (summarized in Fig. 4b).

### Vacuolar pH controls vacuolar metabolite levels

Acidic vacuolar pH is proposed to be essential for the storage of metabolites such as amino acids^1^. We wondered whether the dynamic CDK- and PtdIns(3,5)P_2_-dependent alkalinization and acidification of the vacuolar compartment was sufficient to alter the concentrations of vacuolar metabolites during cell growth. To this end, we followed the fate of the tryptophan analog, 4-cyanotryptophan (4cnTrp) within cells^24^. 4cnTrp differs from tryptophan by two atoms, is excited by 355 nm light, has high quantum yield and its fluorescence intensity is insensitive within biologically relevant pH changes^25^ making it ideal for our purposes.

**Fig. 5:**
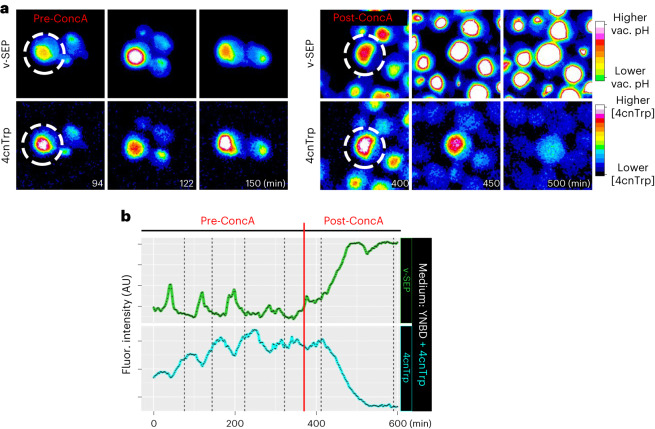
Vacuolar pH acts as a molecular rheostat to control amino acid accumulation in the organelle. **a**, Single frames from timelapse imaging of 4cnTrp and v-SEP before ConcA treatment (pre-ConcA) and after ConcA addition (post-ConcA). **b**, Vacuolar v-SEP and 4cnTrp fluorescence intensity over time are plotted in green and cyan, respectively. The vertical red line denotes the timing of ConcA addition. Note that the v-SEP intensity in this plot is not comparable to the other v-SEP intensity data from the rest of the manuscript as it was generated on a confocal microscope using different exposure times and a different camera (Methods). See Source Data for additional curves. Source data

**Extended Data Fig. 4:**
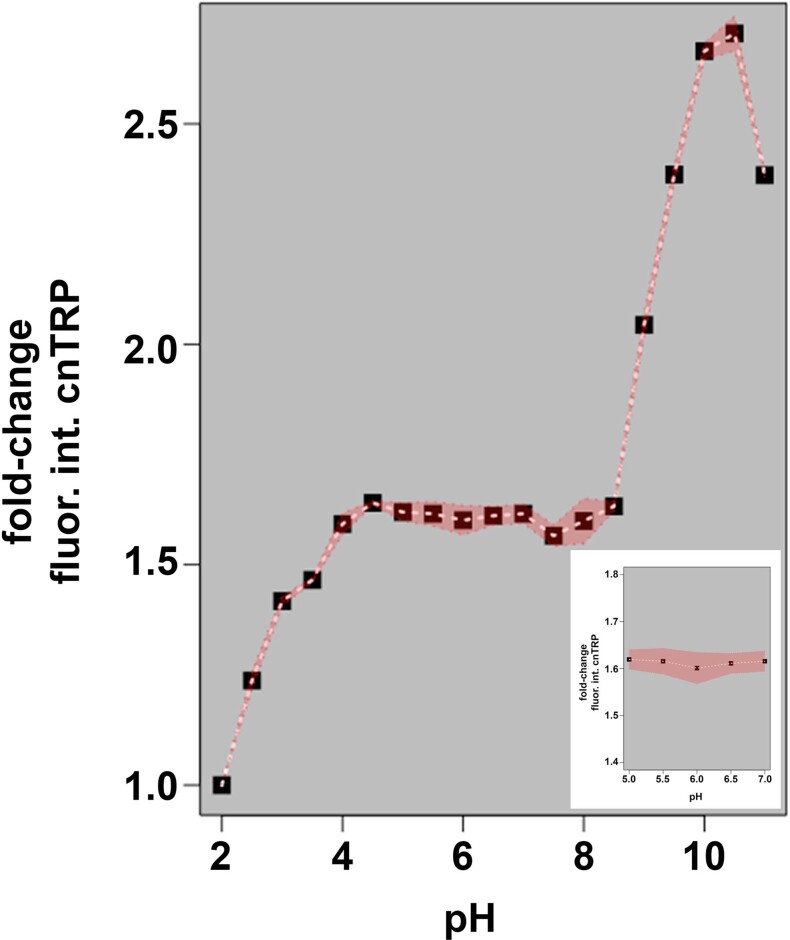
Supporting data for Figure 4. 4cnTrp was added to a buffered pH series^67^ and fluorescence was measured with a CLARIOstar Plus plate reader. The red shaded area is the 95% confidence interval around the mean (dashed line). Source data

We first verified that 4cnTrp, synthesized by an engineered variant of TrpB from *Thermotoga* *maritima*^26^, showed no fluorescence changes in the pH ranges 5.5–7.0 (Extended Data Fig. 4). We then added 4cnTrp to cells as the sole extracellular amino acid source. Imaging 4cnTrp and v-SEP showed that 4cnTrp accumulated in the vacuole and stimulated vacuolar pH oscillations just as tryptophan did (Fig. 5a,b, micrographs and plots: pre-ConcA, and Supplementary Video 11). Notably, 4cnTrp accumulation in the vacuole was oscillatory and increased when the vacuolar pH was most acidic (Fig. 5b, pre-ConcA). This was consistent with the idea that vacuolar pH was essential for the accumulation of vacuolar metabolites. To test whether vacuolar pH was the major determinant of accumulation and retention of 4cnTrp in the vacuole, we treated cells with the v-ATPase inhibitor, ConcA, while imaging v-SEP and 4cnTrp. Inhibition of the v-ATPase led to the rapid efflux of 4cnTrp from the vacuole, concordant with the alkalinization of the organelle (Fig. 5a,b, micrographs and plots: post-ConcA).

During our characterization of vacuolar pH changes, we noticed that there was a distinct change in refractive index and apparent tonicity of the vacuole that occurred simultaneously with the peak of vacuolar alkalinization (Fig. 1e, red arrow, and Supplementary Video 5). This suggests an alteration of the solute concentration within the vacuole^27^ and may indicate an exchange of solutes between the cytoplasm and the vacuole that is coupled with alterations of vacuolar pH. Taken together, these data suggest that vacuolar pH dynamics are of sufficient magnitude to move metabolites out of the vacuole and that the vacuolar pH gradient may be acting as a molecular rheostat that is controlling the concentrations of many stored metabolites in the vacuole.

### Vacuolar pH dynamics coordinates amino acid metabolism

**Fig. 6:**
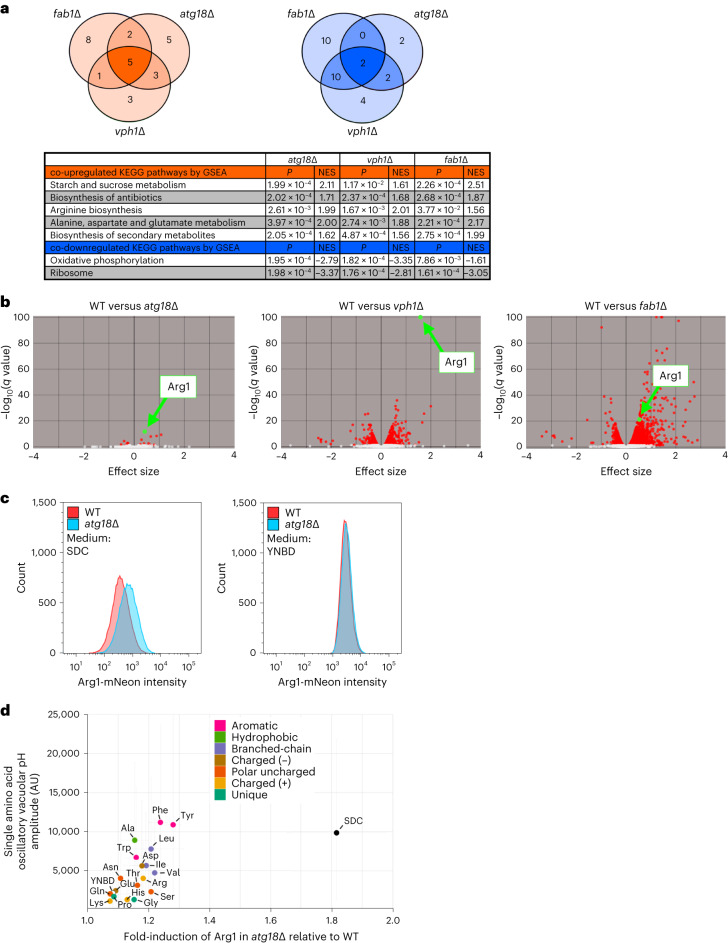
Cells unable to oscillate vacuolar pH downregulate ribosomal and oxidative phosphorylation genes and induce arginine biosynthetic genes. **a**, Venn diagrams of shared co-upregulated (orange) and co-downregulated (blue) KEGG pathways identified by GSEA analysis comparing transcriptomes of *fab1∆*, *atg18∆* and *vph1∆*. KEGG pathways are shown in tabular format with *P* values and normalized enrichment scores (NES) for each mutant. **b**, Volcano plots of transcriptional changes in *atg18∆*, *vph1∆* and *fab1∆* cells compared to wild-type cells. Differentially expressed genes, *q* value > 0.05, are colored in red and Arg1 is highlighted in green. **c**, Histograms of Arg1-mNeon expression analyzed by flow cytometry comparing Arg1 levels in wild-type and *atg18∆* cells in medium containing amino acids (left, SDC, WT $\underline{x}$ = 496.4 AU versus *atg18∆* $\underline{x}$ = 901.0 AU, two-sided *t*-test *P* < 2.2 × 10^−16^) and without amino acids (right, YNBD, WT $\underline{x}$ = 3,235.1 AU versus *atg18∆* $\underline{x}$ = 3,520.6 AU, two-sided *t*-test *P* < 2.2 × 10^−16^). **d**, plot of a single amino acid’s oscillatory vacuolar pH amplitude (from Fig. 2d) versus the fold Arg1-mNeon induction in *atg18∆* cells relative to WT. Error bars represent 95% CI. Source data

To better understand the biological importance of oscillatory vacuolar pH dynamics and metabolite release, we sought to identify a common phenotypic profile among mutants that lacked vacuolar pH oscillations. All the strong vacuolar pH oscillation mutants have pronounced defects in cell growth except for *atg18*∆, the regulator of PtdIns(3,5)P_2_ dynamics associated with the Fab1 complex. To bypass potential pleiotropy due to growth deficits and to narrow in on the phenotypic consequences of blocking vacuolar pH oscillations, we performed RNA-seq to compare the transcriptomes of three strains unable to oscillate vacuole pH each of which have different effects on cellular physiology. We used strains with a single deletion of the Fab1 lipid kinase (*fab1∆*), the negative regulator of the Fab1 complex (*atg18∆*) and the vacuolar specific subunit of the v-ATPase (*vph1∆*). We performed gene set enrichment analysis (GSEA) to identify shared up- and downregulated biochemical pathways^28^ and identified five significant co-upregulated and two co-downregulated KEGG pathways shared between *atg18∆*, *fab1∆* and *vph1∆* strains (Fig. 6a). The genes involved in the co-downregulated pathways consisted of two classes, oxidative phosphorylation and the ribosome. The downregulation of oxidative phosphorylation is consistent with previous reports relating the v-ATPase and the Fab1 complex to mitochondrial function^29^. The downregulation of ribosomal protein genes suggested that cells unable to oscillate vacuolar pH were experiencing proteotoxic stress^30^ and reduced translational capacity^31^.

An examination of the co-upregulated genes suggested that several biochemical pathways related to the production of metabolites were induced (Fig. 6a). First, the upregulation of starch and sucrose metabolism genes was driven largely by genes involved in glycogen metabolism, the transcription of which are controlled by carbon and nitrogen source availability^32^. The last four classes of co-upregulated KEGG pathways all centered around nitrogen metabolism, with the genes *ARG1* and *ARG3* providing a major contribution to each enriched class. Differential expression analysis highlighted the relatively modest global transcriptional changes in *atg18∆* relative to *fab1∆* and *vph1∆*, but shows that Arg1 was one of the most significantly upregulated genes in all three deletion mutants (Fig. 6b). Together, these data suggest that one of the shared phenotypes for cells unable to oscillate vacuolar pH is a perceived nitrogen imbalance and the upregulation of arginine biosynthetic genes.

**Extended Data Fig. 5:**
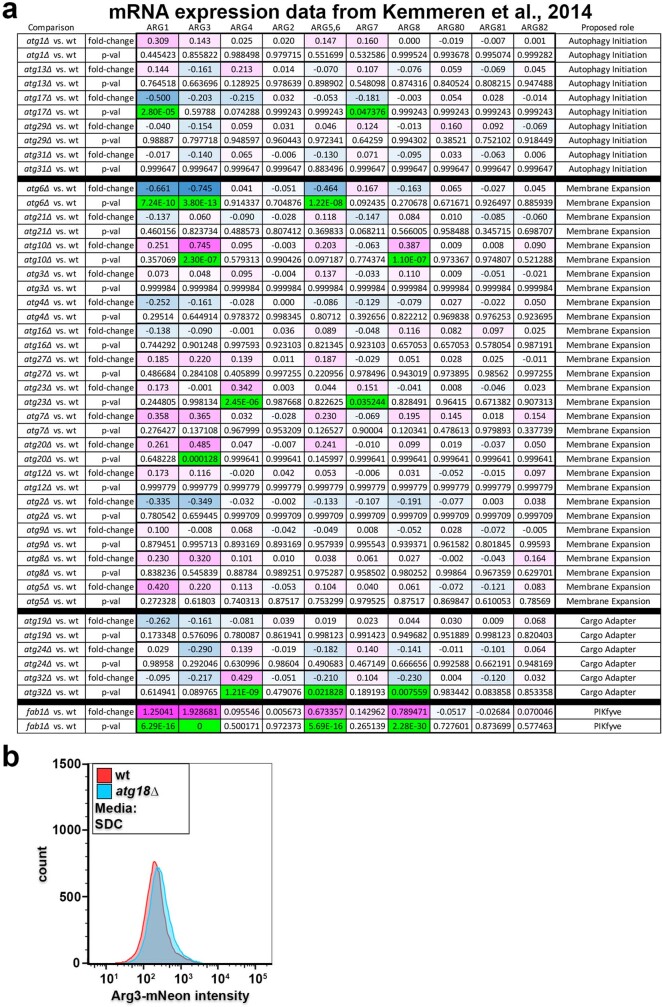
Supporting data for Figure 5. **(a)** mRNA expression profiles for autophagy mutants extracted from Kemmeren et. al, 2014^34^ were subsetted on arginine biosynthetic genes. Data is formatted such that upregulated genes are displayed in shades of magenta and downregulated genes are displayed in shades of blue. Significant expression changes (p-val < 0.05) are highlighted in green. All significance values are from Kemmeren et. al. **(b)** Histograms of Arg3-mNeon expression analyzed by flow cytometry comparing Arg3 levels in wild-type and *atg18∆* cells in media containing amino acids. Wild-type x = 287.7 AU vs. *atg18∆* x = 369.6 AU, two-sided t-test p-val < 2.2 × 10^−16^. Source data

Atg18 is an important component of the autophagic machinery which, together with Atg2, has a key role in autophagosome expansion^33^. We wondered whether defective autophagy might be playing a role in upregulation of *ARG1* and *ARG3 in atg18∆* cells. To explore this, we re-analyzed previously published messenger RNA expression data from 1,484 knockout yeast strains^34^ and extracted mRNA fold changes in autophagy knockout strains relative to wild-type cells, focusing on transcriptional changes in arginine biosynthetic genes. These data show no significant changes in *ARG1* or *ARG3* mRNA in 23 strains lacking key components of the autophagy process (Extended Data Fig. 5a), suggesting that *atg18∆* cells upregulate ARG1 and ARG3, which is independent of defects in autophagy.

The arginine biosynthetic pathway is noted for the direct regulation by arginine at the enzyme level, the transcriptional level and the translational level^35^. The first five steps of arginine biosynthesis take place in the mitochondrion, whereas the final three steps, catalyzed by Arg1, Arg3 and Arg4, take place in the cytoplasm. Arginine’s central role in nitrogen metabolism, its strong accumulation in the vacuole and the upregulation of key cytoplasmic players in arginine biosynthesis led us to wonder whether vacuolar pH oscillations are important for supplying the cytoplasm with arginine under conditions of excess alanine, aromatic, or branched-chain amino acids. To see whether the transcriptional induction of these key cytoplasmic enzymes was recapitulated at the protein level, we tagged ARG1 and ARG3 with mNeon. In particular, *atg18∆* cells were analyzed because of their relatively benign growth phenotype. We found that Arg1 was indeed upregulated ~1.8-fold in *atg18∆* cells grown in conditions where vacuolar pH oscillations were normally induced (Fig. 6c, SDC). A similar, but less pronounced (1.3-fold), upregulation of Arg3 was also observed (Extended Data Fig. 5b). In medium lacking amino acids (YNBD), where Arg1 is already highly expressed, there was only a slight (1.1-fold) upregulation of Arg1 in *atg18∆* cells compared to wild-type cells (Fig. 6c).

We then asked whether the upregulation of Arg1 in *atg18∆* was induced by particular amino acids. We found that, for each single amino acid added, the magnitude of induction of Arg1 in *atg18∆* relative to wild-type cells was correlated with the corresponding oscillatory vacuolar pH amplitude. This analysis revealed a clear correlation in which alanine, aromatic and branched-chain amino acids individually produced the strongest induction of Arg1 (Fig. 6d), while together all amino acids (SDC) produce the maximal induction. This suggests that amino acids that induce Arg1 expression in *atg18∆* are the same that induce vacuolar pH oscillations and that there is an additive effect of all amino acids on Arg1 induction.

From these results we propose that cells are using vacuolar pH oscillations to fine tune their arginine biosynthetic capacity in response to changes in cytoplasmic alanine, branched-chain and aromatic amino acid availability. When faced with the inability to access a vacuolar pool of arginine, cells downregulate ribosomal proteins, reduce their mitochondrial function and attempt to upregulate cytoplasmic arginine biosynthesis.

### Vacuolar pH regulation contributes to cell size control

Emerging evidence suggests that dynamic changes in amino acid levels support changes in protein synthetic capacity as cells transit through the cell cycle^36,37^. Given this, the apparent coordination of arginine biosynthesis with alanine, aromatic and branched-chain amino acid availability (Fig. 6d) and our observation that the vacuole is most alkaline just before cell division (Fig. 1c and Extended Data Fig. 1b), led us to ask whether vacuolar pH oscillations impact aspects of cell cycle regulation. Specifically, we examined the well-established coordination of cell birth size and the time it takes for that newborn cell to commit to a DNA replication event^38,39^. This was tested by determining whether mutant cells that lack the ability to release amino acids from the vacuole during the cell cycle have defects in these coordinated events.

**Fig. 7:**
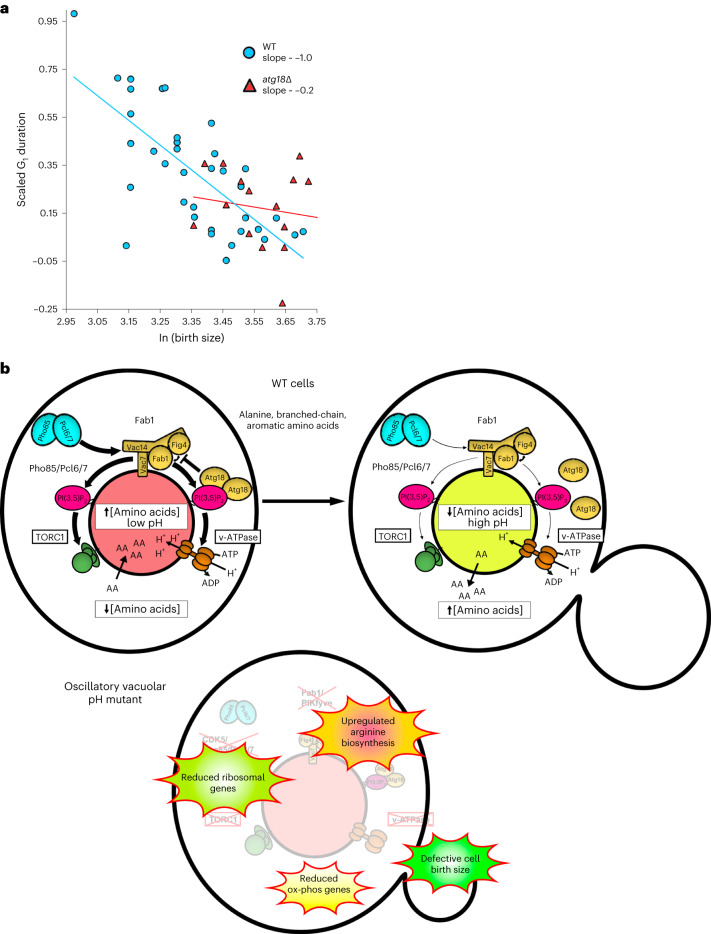
Cell cycle-linked vacuolar pH dynamics contribute to cell size control. **a**, Plot of relative growth in G_1_ versus ln(birth size) in wild-type (blue circles) and *atg18∆* (red triangles). Slope of the linear regression is indicated for each strain in the inset. **b**, A schematic model shows the regulation of vacuolar pH as WT cells grow in the presence of alanine, branched-chain and aromatic amino acids (AAs). In unbudded cells (left), vacuolar pH is more acidic, which drives the accumulation of amino acids in the lumen of the organelle. As cells grow (right), the organelle becomes less acidic and releases amino acids into the cytoplasm. pH regulation requires Pho85/Pcl6/Pcl7-dependent stimulation of the Fab1 complex to produce PtdIns(3,5)P_2_, which recruits Atg18 and reciprocally activates TORC1 and the v-ATPase. During growth, PtdIns(3,5)P_2_ levels at the vacuole drop, which in turn downregulates both TORC1 and v-ATPase leading to an increase in vacuolar pH and efflux of vacuolar stored amino acids. Mutant cells unable to oscillate vacuolar pH during growth (bottom) downregulate ribosomal and mitochondrial genes and upregulate arginine biosynthesis to attempt to maintain homeostasis. These events lead to dysregulated coordination of cell size and timing for the initiation of DNA replication. ox-phos, oxidative phosphorylation. Source data

To measure cell size, we labeled the plasma membrane with mRuby2 fused to the first 28 residues of the palmitoylated plasma membrane-associated phosphatase, Psr1. To minimize phototoxicity over the imaging period we used a custom-built, single-objective lightsheet microscope^40^ to capture full volumetric data as the cells grew exponentially in SDC. We quantified the volume of the daughter cell when it separated from its mother and the growth that the daughter underwent before initiating the formation of a new bud (relative G_1_ duration). The scaled G_1_ duration was negatively correlated with the cell size at birth in wild-type cells (Fig. 7a, blue circles, and Supplementary Video 12; slope ~−1.0, *P* = <1 × 10^−4^). Thus, as previously described^39^, the G_1_-to-S transition in these wild-type yeast cells operated under a size control regime; however, in *atg18∆* cells there was a breakdown in the size control (Fig. 7a, red triangles, and Supplementary Video 13; slope ~−0.2, *P* = 0.49). That is, the slope of the scaled G_1_ duration versus log-transformed cell size at birth was ~−0.2 in *atg18∆* instead of ~−1.0 as seen in wild-type cells (*P* = 2.3 × 10^−2^, *F*-test, wild-type versus *atg18∆* slope). This suggests that proper size control in yeast relies, at least in part, on the cell cycle regulated coordination of amino acid pools generated by the dynamic control of vacuolar pH.

## Discussion

Microorganisms and many metazoan cell types are tuned to take up nutrients when available and to coordinate this with rapid growth. As a result, homeostatic mechanisms have evolved to maintain appropriate levels of cellular metabolites despite being faced with fluctuating nutrient availability and/or cellular demand (for example growth and protein secretion). Recent work in tissue culture with mammalian cells^41^ identified that amino acid uptake and storage within lysosomes during nutrient-replete conditions was critical to support translation during nutrient starvation; however, it is less clear whether amino acid compartmentalization in actively growing cells is important^42^.

The present study reveals a new and unexpected layer of regulation of vacuolar pH and amino acid homeostasis in unstressed, growing cells. We find that cells growing in the presence of alanine, branched-chain or aromatic amino acids undergo regular vacuolar alkalinization and reacidification events once per cell cycle (Fig. 7b). We propose that cells possess cytoplasmic amino acid-sensing mechanisms that integrate diverse intra- and extracellular metabolic cues (glycolytic versus respiratory carbon source and amino acid type; see Fig. 2) to sequester and then mobilize amino acids through dynamic modulation of vacuolar pH in an attempt to maintain amino acid homeostasis during cell growth. Consistent with a role for carbon source metabolism in amino acid homeostasis, it has been reported that carbon catabolite repression is functionally intertwined with amino acid metabolism through TORC1 signaling^43^.

Our data suggest that cells use internal pools of stored amino acids as a buffer to support multiple cell systems including translation, mitochondrial function and cell size. We propose that if cells are unable to dynamically regulate vacuolar pH in the presence of certain extracellular amino acids they may be unable to liberate vacuolar pools of amino acids such as arginine. This deficit leads to reduced translational capacity through downregulation of ribosomal transcript levels, reduced mitochondrial function by downregulating oxidative phosphorylation genes (Fig. 6a) and consequently dysregulated coordination of cell size and timing of G_1_ (see Fig. 7a). The maintenance of translational capacity and/or mitochondrial function during cell growth may impact the translation of key cell cycle regulatory proteins such as the important regulator of the G_1_-to-S transition, Cln3 or through the regulation of other cell cycle steps that sense metabolite/amino acid levels of the cell^44–46^.

In addition, the sequestration of amino acids in the vacuole may be important in another way for cellular homeostasis: prevention of amino acid imbalance during nutrient abundance in the environment. Such imbalances can impact a number of cellular processes. Amino acid imbalances can lead to transfer RNA mischarging and consequently mistranslation^47^. Furthermore, amino acid concentration and codon optimality are a major driving source of mRNA stability^48^. Amino acid imbalances can also alter flux through anabolic pathways due to feed-forward inhibition^35^. Recently it was shown that excess cysteine in the cytoplasm leads to collapse of iron–sulfur cluster biogenesis and mitochondrial health during the aging process^4^.

The detailed mechanism of how vacuolar acidity is lost and then regained during cell growth is likely to be mechanistically complex. Our data show a role for the v-ATPase, TORC1, Pho85 (or CDK5) and PtdIns(3,5)P_2_ generation in regulating vacuolar pH dynamics during the cell cycle (Fig. 7b). Our observation that the PtdIns(3,5)P_2_ binding protein and regulator of the Fab1 complex, Atg18, is dynamically recruited to the vacuolar limiting membrane suggest that the key regulatory lipid species PtdIns(3,5)P_2_ may be oscillating in growing cells (Fig. 4a). While compromising the core Fab1 kinase complex leads to lower PtdIns(3,5)P_2_ levels and compromising Atg18 function leads to higher PtdIns(3,5)P_2_ levels, both individual perturbations block vacuolar pH oscillations. This suggests that the appropriate cycling of PtdIns(3,5)P_2_ is essential to coordinate different phases of vacuolar acidification and alkalinization, though it is also possible that Atg18 may be playing a more direct role in regulating amino acid release from the vacuole. The importance of PtdIns(3,5)P_2_ in regulating the v-ATPase and TORC1 and the role of the nutrient-responsive CDK Pho85 for modulating Fab1 activity, suggests there is a rich regulatory network that will require further mechanistic dissection. Moreover, the fact that all these biological pathways are highly conserved raises the possibility that lysosomal pH and lysosomal metabolite mobilization may be dynamically regulated in other eukaryotes. In particular, we expect these dynamics to be important in conditions where an increased demand for protein synthesis or other aspects of cellular metabolism provides an advantage for rapid cell growth, such as embryogenesis, cancer and the activation of quiescent stem cells.

Recent work suggests that amino acid biosynthetic pathways are optimized to particular stages of the cell cycle to support the translation of proteins with skewed amino acid compositions^37^. We propose that yeast are also using vacuolar pH regulation and amino acid mobilization to augment these attempts to maintain translational capacity during the cell cycle (Fig. 7b). Remarkably, our results suggest that the inability to oscillate vacuolar pH results in a dysregulation of the well-established control of size homeostasis in yeast. While the exact mechanism of size control remains controversial^36,44,49–51^, our data suggest that dynamic vacuolar pH regulation and its potential impact on cytoplasmic pH^52^ may be playing an important role.

Collectively, this work identifies a new model for dynamic regulation of the metabolite storage capacity of an organelle at the center of many critical cellular pathways. Given the importance and high degree of conservation between humans and yeast in all the pathways identified in the work described here, it is worth noting that mutations in the conserved Fab1, v-ATPase and TORC1 complexes all have impacts on human health^53–55^, raising the possibility that defects in the dynamics of amino acid storage in the lysosome-like vacuole might underlie aspects of the mechanisms of initiation or progression of a variety of human diseases.

## Methods

### Strains, plasmids, growth medium and pharmacological treatments

#### Yeast strains

All strains used in this study are shown in Supplementary Table 1. All prototrophic strains are derived from the DHY background^56^. All knockout and gene-tagged strains were made in a diploid DHY parent strain and haploid strains were generated by sporulation, tetrad dissection and verification of the segregation of the appropriate markers. All deletion strains were made using the dominant drug resistance markers described by the Knop group^57^. c-Terminal mNeon-tagged proteins were made by amplifying an integration cassette from pKT127-mNeonGreen. Strains harboring reporters for monitoring vacuolar pH were made by integrating the construct at the HO locus as described^58^ using the plasmids designated as ‘pHO’ below. Strains harboring genomically encoded mCherry-Atg18 were made by amplifying a PCR fragment containing the NatMX cassette followed by the ADH1 promoter region and mCherry. The entire fragment was integrated at the 5′ end of the native Atg18 locus and verified by PCR. Strains expressing Psr1-mRuby2 were made by amplifying plasmid pADH1pr-PSR1-mRuby2 with primers that allow the insertion of the construct into an empty region of chromosome I (199,458–199,459) in a *ura3*^−^ DHY WT strain. Strains harboring *fab1-21a* and *vac7-7a* were made by amplifying a PCR fragment containing the NatMX cassette following the mutant variants of each gene. Each fragment was integrated at the native Fab1 or Vac7 locus, respectively.

#### Plasmids

pKT127-mNeonGreen was made by amplifying mNeonGreen with PacI and AscI sites and replacing eGFP in pKT127-eGFP^59^.

pHO-KanMX-TEF1prom-CPYleader-v-SEP-mCherry was made by first creating a donor plasmid that contained the first 50 amino acids of CPY (Prc1) introduced by annealing complementary oligonucleotides flanked by XbaI and SpeI sites and v-SEP flanked by SpeI and XhoI sites. The CPYleader-v-SEP fragment was then amplified by PCR to contain flanking BamHI and XhoI sites and cloned into p416TEF^60^ using those sites. The TEFprom-CPYleader-v-SEP-CYCterm was then amplified and cloned into pHO-KanMX^58^ using SmaI. Yeast codon optimized mCherry (yemCherry) was then cloned downstream and in frame of v-SEP using HiFi DNA assembly (NEB) according to the manufacturer’s protocol.

pHO-NatMX-TEF1prom-CPYleader-v-SEP-mScarlet was made by replacing the KanMX cassette of pHO-KanMX with NatMX using a fragment of DNA digested from pFA6a-NatMX-NT2^57^ flanked by AscI and EcoRI. Yeast codon optimized mScarlet (yemScarlet) was then cloned downstream and in frame of v-SEP using HiFi DNA assembly (NEB) according to the manufacturer’s protocol.

pUC19-ADH1pr-mCherry-Atg18 was made by first amplifying the NatMX-NT2 cassette from pFA6a-NatNT2^57^ flanked by EcoRI and SacI restriction enzyme sites and cloning it into pUC19. The rest of the construct was constructed sequentially such that the final construct contained the ADH1 promoter flanked by SacI and XbaI sites, the yemCherry fluorophore flanked by XbaI and SalI sites and the *ATG18* ORF flanked by SalI and SacI. The yemCherry and *ATG18* ORF are separated by a 6× glycine linker that was introduced by PCR.

pADH1pr-PSR1-mRuby2 was made by replacing ratiometric phluorin (RMP) in pADH1pr-PSR1-RMP^61^ with mRuby2 using the flanking PacI and AscI sites. This plasmid is marked by a URA3 selection cassette.

Plasmids containing *fab1-21a* or *vac7-7a* with their native promoter and terminator flanked by NotI and SbfI sites were synthesized by Twist Bioscience and cloned into pUC19. NatMX was amplified with flanking SbfI sites and cloned downstream.

All oligos used to construct yeast strains and plasmids are listed in Supplementary Table 2.

All constructs were verified by Sanger sequencing.

#### Growth medium

SDC contained SC supplement mix (Sunrise) YNB + nitrogen (ammonium sulfate) mix (Sunrise) and 2% dextrose, pH 4.5. This medium was consistently pH ~4.5 without any additions. YNBD contains YNB + nitrogen (ammonium sulfate) mix (Sunrise) and 2% dextrose and was adjusted to pH ~4.5 with the addition of KOH to match SDC. All individual amino acid addback medium used 85.6 mg l^−1^ amino acids except for leucine which was 173.4 mg l^−1^. All individual amino acid addback medium was adjusted to pH ~4.5. SCEG contained SC supplement mix (Sunrise) YNB + nitrogen (ammonium sulfate) mix (Sunrise), 2% glycerol and 3% ethanol, pH 4.5. All experiments and yeast cell growth were performed at room temperature.

#### Pharmacological treatments

Rapamycin (Fisher) was resuspended to 1 mg ml^−1^ in 90% ethanol/10% Tween-20 and used at a working concentration of 1 µg µl^−1^ in SDC. Concanamycin A (MiliporeSigma) was resuspended to 1 mg ml^−1^ in dimethylsulfoxide (DMSO) and used at a working concentration of 1 µg ml^−1^ in SDC. For all treatments, cells were grown in microfluidics devices for 9 h in untreated medium and then flow of drug containing-medium was automatically initiated by the microfluidics system described below.

### Microfluidics

Cells were cultured in the appropriate medium for 24 h and then diluted to an appropriate OD_600_ such that after 12–16 h of growth the cell density was between OD_600_ 0.05–0.1 the next day. This served two purposes: to achieve the appropriate cell-loading density and to limit medium acidification in the starter culture. Cells were loaded into a CellASIC ONIX plate for haploid yeast cells (Y04) and the appropriate medium was flowed over the cells (21 kPa) using a CellASIC ONIX2 microfluidic system (Millipore) for 1,000 min.

For the data presented in Fig. 1d, a custom microfluidic platform^9^ was used to image v-SEP and mCherry fluorescence every 5 min for ~1,000 min.

### Microscopy

#### Wide-field fluorescence imaging in CellASICs devices

v-SEP and mCherry fluorescence was recorded on a Nikon Ti equipped with an Andor Neo sCMOS camera, standard FITC/TRITC filter sets, a Sola 6 LED light source (Lumencor) and a ×60 objective. All data were acquired as single medial focal plane images with fluorescence and brightfield data acquired every 2 min using a 50-ms exposure with 12% incident light intensity for v-SEP and 150-ms exposure with 12% incident light intensity for mCherry.

#### FLIM imaging of v-mScarlet

Cells expressing v-mScarlet were grown in SDC and fluorescence lifetime data were collected using a Leica SP8 point-scanning confocal microscope equipped with an LSM/FLIM module (PicoQuant) for time-correlated single photon counting. To avoid phototoxicity, we imaged continuously at very low laser intensity. We used a HC PL APO CS ×100/1.4 NA objective, illumination from a tunable white light laser at 40 MHz with a 561 excitation filter, a pixel size of 151.96 nm and a 97.65-μs dwell time. The 256 × 256-pixel images were collected using an open pinhole on a HyD SMD detector with an emission window of 581–750 nm. Each image required 26 s to acquire. We used Picoquant software to display the average photon arrival time, binning four images in time and applying two-dimensional smoothing of 500 nm.

#### BCECF imaging in CellASICs devices

Cells were grown in SDC as noted above and treated with 5 µM BCECF (Thermo Fisher). BCECF fluorescence was recorded on a Leica DMi8 equipped with a pco.edge 4.2 camera (pco), a SpectraX LED light source (Lumencor) and a ×60 objective. Both pH-sensitive and insensitive BCECF fluorescence was obtained using a 540/21 emission filter and a FF444/520/590-Di01 dichroic mirror (Semrock). pH-insensitive fluorescence was obtained illuminating the sample with a 427/10 bandpass filter and pH-sensitive fluorescence used a 540/21 bandpass filter. For both fluorescence signals, incident light was attenuated to 30% power and an exposure times of 45 ms was used.

#### 4cnTrp imaging by confocal microscopy

Cells were grown in YNBD + 4cnTrp (85.6 mg l^−1^) as described above. v-SEP and 4cnTrp fluorescence was recorded on a Leica DMi8 equipped with an ORCA-Flash4.0 camera (Hamamatsu), standard FITC/DAPI filter sets, a Sola light engine (Lumencore) and a ×100 HC PL Apo TIRF objective. All data were acquired as single medial focal plane images with fluorescence and brightfield data acquired every 2 min using a 25-ms exposure with 15% incident light intensity for v-SEP and 200-ms exposure with 15% incident light intensity for 4cnTrp.

#### Wide-field fluorescence imaging of v-SEP in open chambered slides

Cells were grown in SDC as described above and allowed to settle for 5 min on a 24 × 60 mm no. 1 glass coverslip that was coated with 0.1 mg ml^−1^ concanavalin A (Fisher). A ring of vacuum grease was used to make an open well around the cells. After settling, unattached cells were washed away with fresh medium and an excess of fresh medium was added to the well (~500 µl). v-SEP fluorescence was recorded on a Nikon Ti equipped with an Andor Neo sCMOS camera, standard FITC filter sets, a Sola 6 LED light source (Lumencor) and a ×60 objective. All data were acquired as single medial focal plane images with fluorescence and brightfield data acquired every 2 min using a 50-ms exposure with 12% incident light intensity for v-SEP.

#### Single-objective lightsheet microscopy of Psr1-leader-mRuby2

A SOLS microscope with a Nikon ×100 1.35 silicone primary objective was used to collect single color volumetric time-series data (see elsewhere^40^ for microscope configuration details). For fluorescence excitation and emission, a 561-nm laser (Coherent OBIS LS 80 mW) was used in conjunction with a quad-band dichroic (Chroma ZT405/488/561/640rpcv2) and longpass emission filter (Chroma LP02-561RU). In total, 240 volumes were taken per dataset at 300-s intervals with a voxel size of (116 × 116 × 469) nm^3^ (108 slices per volume and 1,060 × 548 pixels per slice in practice). Each image slice was taken with a 100-ms exposure and relatively low laser power to minimize photodamage (5% setting + Thorlabs NDE10A absorptive filter).

### RNA-seq

Duplicate cultures on two separate days (four replicates total) were grown in the appropriate medium to OD_600_ = 0.05, collected by filtration onto MF Membrane filters (Millipore, HAWP02500) and snap-frozen in liquid nitrogen. Frozen cells were processed as described elsewhere^62^. Briefly, cells were resuspended in 200 µl lysis buffer (10 mM Tris, pH 8.0, 0.5% SDS and 10 mM EDTA) and separated from the filter disks. Then, 200 µl Acid Phenol, pH 4.3, was added and the samples were vortexed for 30 s. The samples were incubated at 65 °C for 1 h in a Thermomixer with intermittent shaking (2,000 r.p.m. for 1 min every 15 min). Then, 400 µl 100% ethanol was added and the RNA was purified using the Direct-zol RNA Miniprep Plus kit (Zymo Research) according to the manufacturer’s protocol, including the DNase digestion step. RNA integrity was assessed using an Agilent Bioanalyzer and one WT sample was eliminated from analysis because the RNA was of insufficient quality.

RNA-seq analysis was executed and visualized using an in-house, web-based platform, consisting of the following steps. Sequencing quality control was performed using FastQC (v.0.11.5). Transcript expression was then quantified using Salmon^63^ (v.0.9.1) in pseudo-alignment mode, without adaptor trimming, producing transcript-per-million estimates, using the Ensembl (SacCer3) transcriptome. Differential expression analysis was performed in R using the Sleuth package^64^ (v.0.29.0), producing gene-level effect sizes and *q* values for *atg18∆*, *vph1∆* and *fab1∆* versus WT. The list of genes ranked by *q* value was then used to perform GSEA using the fgsea package^65^ (v.1.4.1). RNA-seq data are deposited at the Gene Expression Omnibus (GSE236913).

### Flow cytometry

**Extended Data Fig. 6:**
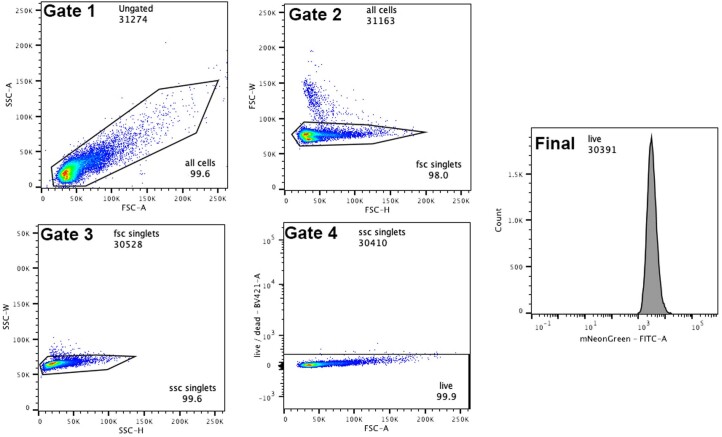
Representative gating strategy for FACS data. A representative gating strategy for one sample (wild-type cells expressing Arg1-mNeon growing in YNBD) used for all FACS analysis presented in this manuscript.

Cells were grown overnight in the appropriate medium, diluted in fresh medium to OD_600_ = 0.03–0.05 and grown for 2–3 h. Cells were stained in medium with 1 µM SYTOX Blue (Thermo Fisher) for 5 min and then analyzed on a BD LSRFortessa X-20 Cell Analyzer equipped with the appropriate laser lines and emission filter sets. FACS data were collected with FACSDiva software (v.9.0). At least 30,000 cells per growth medium were analyzed from several separate days. A representative gating strategy for one sample (WT cells expressing Arg1-mNeon growing in YNBD) is presented in Extended Data Fig. 6. Live cells were gated using the SYTOX Blue signal (gate 4, Extended Data Fig. 6). All cytometry data were analyzed by FlowJo (v.10.8.1). Two-sided *t*-tests were performed in R (v.4.1.2) and normality of data was assumed due to the large sample size.

### Microscopy data analysis and measurements of v-SEP pH sensitivity

#### Population level oscillatory vacuolar pH amplitude estimation

Data from microfluidics experiments were processed with Fiji (1.53k) and the TrackMate plugin (v.6.0.3). To process data for analysis in TrackMate, timelapse data were background subtracted using rolling ball subtraction with a radius of 50 pixels and then registered using the StackReg^66^ plugin. TrackMate was then used to segment vacuoles (LoG detector: estimated blob diameter of 27 pixels, threshold of 0.3) and track their fluorescence in time (Linear Motion Lap tracker: initial search radius of 25 pixels, search radius of 20 pixels)

The TrackMate outputs were analyzed using custom-built R scripts. In brief, individual fluorescence intensity traces were selected such that there were at least 500 min of data, the data were detrended using a time-series linear model (forecast::tslm), PSD analysis was performed (Genecycle::periodogram) on the detrended data and the maximum amplitude and the period of the maximum amplitude was noted for each trace. This process was iterated over all of the data from any particular growth condition. The median maximum amplitudes and 95% CI from each growth condition or mutant were calculated using the datawizard::describe_distribution (data, centrality = ‘median’, ci = TRUE)) function in R (v.4.1.2 (2021-11-01)).

#### pH sensitivity of v-SEP in situ

BY4741 yeast expressing v-SEP from a p416 plasmid with a GAP promoter and CYC1 terminator were grown overnight in 10 ml SC medium without uracil. Samples were spun down, washed and resuspended in 500 µl water. Then, 10 µl of the resuspension were combined with 90 µl Carmody^67^ buffer at different pH in a 384-well plate and permeabilized using 1 µl of 10% digitonin in DMSO. Excitation spectra were collected in a SpectraMax i3x spectrophotometer from below the sample.

#### pH sensitivity of 4cnTrp

4cnTrp was added to a buffered pH series^67^ in glass-bottomed plates (Corning) and fluorescence (Ex. 360/20, Em. 460/30) was measured with a CLARIOstar Plus plate reader from the top of the plate.

#### BCECF pH calibration in situ

BCECF calibration curves were obtained by permeabilizing yeasts in 50 mM MES, 50 mM Hepes, 50 mM KCl, 50 mM NaCl, 0.2 M ammonium acetate, 10 mM NaN_3_, 10 mM 2-deoxyglucose and 50 µM FCCP. The pH of the buffer was adjusted in increments of 0.5 pH units and images were acquired using the same microscope acquisition settings used for the timelapse described above.

#### Cell size determination using Psr1-mRuby2

3D volumetric data were maximum intensity projected and the cell’s long axis and short axis were measured after separating from its mother. Cell volume was calculated using the formula for a volume of an ellipsoid V = (4/3) × (long axis) × (short axis)^2^. The scaled G_1_ duration was calculated as elsewhere^68^. Statistical tests for significance of the slope of the linear regression and comparison of WT and *atg18∆* data were determined using GraphPad Prism 9.

### Statistics and reproducibility

No statistical method was used to predetermine sample size and no data were excluded from the analyses. All statistical tests used for analysis are included in the Methods or in the Source Data files for each figure. Data were highly reproducible both at the biological and technical levels.

### Reporting summary

Further information on research design is available in the Nature Portfolio Reporting Summary linked to this article.

## Supplementary information

Reporting Summary

Supplementary YouTube links

Supplementary Videos 1–8 **Supplementary Video 1. v-SEP-mCherry fluorescence in cells grown in SDC**. v-SEP (left), mCherry (middle) intensity over time in cells growing in SDC in CellASICs devices imaged every 2 min. Merged signals are shown on the right (v-SEP in green and mCherry in magenta). **Supplementary Video 2. v-SEP fluorescence in cells grown in SDC**. v-SEP fluorescence (left) over time plotted (right) from vacuoles from two representative cells growing in SDC highlighted with orange and blue circles. **Supplementary Video 3. v-SEP fluorescence in cells grown in batch and imaged after settling onto coverglass**. Timelapse data of v-SEP fluorescence in cells adhered to a glass coverslip directly from a batch grown SDC culture. **Supplementary Video 4. Fluorescence lifetime of v-mScarlet in cells grown in SDC**. Fluorescence lifetime imaging of vacuolar targeted mScarlet growing in SDC. Fluorescence lifetimes are pseudo-colored and overlaid on brightfield images with longer lifetimes represented in magenta and shorter lifetimes in green, corresponding to higher and lower pH respectively. **Supplementary Video 5. BCECF fluorescence in cells grown in SDC**. Timelapse data show brightfield and the ratiometric signal of vacuolar accumulated BCECF in cells growing in SDC. **Supplementary Video 6. v-SEP fluorescence in cells grown in YNBD**. v-SEP fluorescence (left) over time plotted (right) from vacuoles from two representative cells growing in YNBD (defined medium with glucose but without amino acids) highlighted with orange and blue circles. **Supplementary Video 7. v-SEP fluorescence in cells grown in SCEG**. Timelapse data show v-SEP fluorescence in cells growing in SCEG (defined medium with the respiratory carbon sources glycerol/ethanol and amino acids). **Supplementary Video 8. v-SEP fluorescence in wild-type cells treated with rapamycin**. Timelapse data show v-SEP fluorescence in cells growing in SDC pre-rapamycin treatment and post-rapamycin treatment.

Supplementary Videos 9–13 **Supplementary Video 9. v-SEP fluorescence in wild-type cells treated with ConcA**. Timelapse data show v-SEP fluorescence in cells growing in SDC pre-ConcA treatment and post-ConcA treatment. **Supplementary Video 10. mCherry-Atg18 and v-SEP fluorescence in wild-type cells grown in SDC**. Timelapse data show mCherry-Atg18 (left) and v-SEP (right) fluorescence in cells growing in SDC. **Supplementary Video 11. 4cnTrp and v-SEP fluorescence in wild-type cells grown in YNBD+4cnTrp**. Timelapse data show 4cnTrp fluorescence (left) and v-SEP fluorescence (right) in cells growing in SDC pre-ConcA treatment and post-ConcA treatment. **Supplementary Video 12. Psr1-mRuby2 fluorescence in wild-type cells imaged by single-objective lightsheet microscopy**. Maximum intensity projections of full volumetric timelapse data of Psr1-mRuby2 fluorescence in wild-type cells grown in SDC. **Supplementary Video 13. Psr1-mRuby2 fluorescence in** ***atg18∆*** **cells imaged by single-objective lightsheet microscopy**. Maximum intensity projections of full volumetric timelapse data of Psr1-mRuby2 fluorescence in *atg18∆* cells grown in SDC.

Supplementary Table 1 Strains used in this study.

Supplementary Table 2 Oligonucleotides used in this study.

## Source data

Source Data Fig. 1 Source and additional data items supporting Fig. 1.

Source Data Fig. 2 Source and additional data items supporting Fig. 2.

Source Data Fig. 3 Source and additional data items supporting Fig. 3.

Source Data Fig. 4 Source and additional data items supporting Fig. 4.

Source Data Fig. 5 Source and additional data items supporting Fig. 5.

Source Data Fig. 6 Source and additional data items supporting Fig. 6.

Source Data Fig. 7 Source and additional data items supporting Fig. 7.

Source Data Extended Data Fig./Table 1 Source and additional data items supporting Extended Fig. 1.

Source Data Extended Data Fig./Table 2 Source and additional data items supporting Extended Fig. 2.

Source Data Extended Data Fig./Table 3 Source and additional data items supporting Extended Fig. 3.

Source Data Extended Data Fig./Table 4 Source and additional data items supporting Extended Fig. 4.

Source Data Extended Data Fig./Table 5 Source and additional data items supporting Extended Fig. 5.

## Data Availability

Beyond what is available in the manuscript, all data (raw and processed) and analysis tools or custom scripts will be provided by the corresponding authors upon reasonable request. Source data are provided with this paper.
