# Supplementary material for: Cell cycle-linked vacuolar pH dynamics regulate amino acid homeostasis and cell growth
Source: Nat Metab. 2023 Aug 28;5(10):1803–19. doi: 10.1038/s42255-023-00872-1 (PMC10590757; doi:10.1038/s42255-023-00872-1)
Supplement: Supplementary file 1 — Reporting Summary [file 42255_2023_872_MOESM1_ESM.pdf]

nature portfolio

Corresponding author(s): Voytek Okreglak and Daniel Gottschling
Last updated by author(s): July 14, 2023

Reporting Summary

Nature Portfolio wishes to improve the reproducibility of the work that we publish. This form provides structure for consistency and transparency in reporting. For further information on Nature Portfolio policies, see our Editorial Policies and the Editorial Policy Checklist.

Statistics

For all statistical analyses, confirm that the following items are present in the figure legend, table legend, main text, or Methods section.

n/a	Confirmed	
☐	☒	The exact sample size (*n*) for each experimental group/condition, given as a discrete number and unit of measurement
☒	☐	A statement on whether measurements were taken from distinct samples or whether the same sample was measured repeatedly
☐	☒	The statistical test(s) used AND whether they are one- or two-sided<br>*Only common tests should be described solely by name; describe more complex techniques in the Methods section.*
☐	☒	A description of all covariates tested
☐	☒	A description of any assumptions or corrections, such as tests of normality and adjustment for multiple comparisons
☐	☒	A full description of the statistical parameters including central tendency (e.g. means) or other basic estimates (e.g. regression coefficient) AND variation (e.g. standard deviation) or associated estimates of uncertainty (e.g. confidence intervals)
☐	☒	For null hypothesis testing, the test statistic (e.g. *F*, *t*, *r*) with confidence intervals, effect sizes, degrees of freedom and *P* value noted<br>*Give P values as exact values whenever suitable.*
☒	☐	For Bayesian analysis, information on the choice of priors and Markov chain Monte Carlo settings
☒	☐	For hierarchical and complex designs, identification of the appropriate level for tests and full reporting of outcomes
☐	☒	Estimates of effect sizes (e.g. Cohen's *d*, Pearson's *r*), indicating how they were calculated

Our web collection on statistics for biologists contains articles on many of the points above.

Software and code

Policy information about availability of computer code

Data collection	µManager (ver. 2.0) was used to collect all wide-field microscopy data, custom code (https://github.com/amsikking/SOLS_microscope) was used to collect SOLS data, PcoQuant (SymPhoTime 64) was used to collect fluorescence lifetime data FACS data was collected with FACSDiva software (ver. 9.0)
Data analysis	Fiji (1.53k) and the Fiji plugin TrackMate (v6.0.3) was used to extract time-series data from microscopy images. R (ver. 4.1.2) was used for analysis of that time-series data. R (ver. 4.1.2) was also used to analyze FACS data. Prism (ver. 9) was used to analyze SOLS data. RNA-seq data was analyzed using FastQC (v0.11.5), Salmon (v0.9.1) and Sleuth (v029.0)

For manuscripts utilizing custom algorithms or software that are central to the research but not yet described in published literature, software must be made available to editors and reviewers. We strongly encourage code deposition in a community repository (e.g. GitHub). See the Nature Portfolio guidelines for submitting code & software for further information.

Data

Policy information about availability of data

All manuscripts must include a data availability statement. This statement should provide the following information, where applicable:

- Accession codes, unique identifiers, or web links for publicly available datasets
- A description of any restrictions on data availability
- For clinical datasets or third party data, please ensure that the statement adheres to our policy

Beyond what is available in the manuscript, all data (raw and processed) and analysis tools will be provided by the corresponding authors upon reasonable request.

Human research participants

Policy information about studies involving human research participants and Sex and Gender in Research.

Reporting on sex and gender	N/A
Population characteristics	N/A
Recruitment	N/A
Ethics oversight	N/A

Note that full information on the approval of the study protocol must also be provided in the manuscript.

Field-specific reporting

Please select the one below that is the best fit for your research. If you are not sure, read the appropriate sections before making your selection.

☒ Life sciences ☐ Behavioural & social sciences ☐ Ecological, evolutionary & environmental sciences

For a reference copy of the document with all sections, see nature.com/documents/nr-reporting-summary-flat.pdf

Life sciences study design

All studies must disclose on these points even when the disclosure is negative.

Sample size	No sample-size calculations were performed. Generally more than 50 cell traces are used for analysis for each mutant or environmental condition. This cutoff was chosen because of the observed consistency and reproducibility of the phenotypes analyzed.
Data exclusions	After determination of RNA integrity using an Agilent Bioanalyzer one wild-type sample was eliminated from analysis because the RNA was of insufficient quality. This is stated in the material and methods in the manuscript.
Replication	All data was reproducible and consistent between experimental conditions and mutants. All experiments were performed at least 3 times.
Randomization	Samples were randomized in their position in the CellASICS microfluidics device from experiment to experiment to minimize potential technical artifacts. Amino acid add back experiments were pseudo-randomized by physico-chemical property by performing the analysis by alphabetical order of amino acid.
Blinding	Blinding was not generally applicable for these studies because there was limited preconceived bias for the experimental outcome.

Reporting for specific materials, systems and methods

We require information from authors about some types of materials, experimental systems and methods used in many studies. Here, indicate whether each material, system or method listed is relevant to your study. If you are not sure if a list item applies to your research, read the appropriate section before selecting a response.

Materials & experimental systems

n/a	Involved in the study	
☒	☐	Antibodies
☐	☒	Eukaryotic cell lines
☒	☐	Palaeontology and archaeology
☒	☐	Animals and other organisms
☒	☐	Clinical data
☒	☐	Dual use research of concern

Methods

n/a	Involved in the study	
☒	☐	ChIP-seq
☐	☒	Flow cytometry
☒	☐	MRI-based neuroimaging

Eukaryotic cell lines

Policy information about cell lines and Sex and Gender in Research

Cell line source(s)	All strains used here are described in the material and methods and in Supplemental Table 2.

Authentication | All founder wild-type strains were sequenced.
Mycoplasma contamination | N/A
Commonly misidentified lines (See ICLAC register) | N/A

Flow Cytometry

Plots

Confirm that:

- [x] The axis labels state the marker and fluorochrome used (e.g. CD4-FITC).
- [x] The axis scales are clearly visible. Include numbers along axes only for bottom left plot of group (a 'group' is an analysis of identical markers).
- [x] All plots are contour plots with outliers or pseudocolor plots.
- [x] A numerical value for number of cells or percentage (with statistics) is provided.

Methodology

Sample preparation | This is describes in the material and methods

Instrument | BD LSRFortessa X-20 Cell Analyzer

Software | FACS data was collected with FACSDiva software (ver. 9.0)

Cell population abundance | At least 30,000 cells per growth medium were analyzed from several separate days.

Gating strategy | Live cells were gated using SYTOX Blue signal.

- [x] Tick this box to confirm that a figure exemplifying the gating strategy is provided in the Supplementary Information.
