# Supplementary material for: Cell cycle-linked vacuolar pH dynamics regulate amino acid homeostasis and cell growth
Source: Nat Metab. 2023 Aug 28;5(10):1803–19. doi: 10.1038/s42255-023-00872-1 (PMC10590757; doi:10.1038/s42255-023-00872-1)
Supplement: Supplementary file 2 — Supplementary YouTube links [file 42255_2023_872_MOESM2_ESM.pdf]

Movie title	Video link
Okreglak Nat Metab 2023 video 1	https://youtu.be/FxD6yZapHdM
Okreglak Nat Metab 2023 video 2	https://youtu.be/oa_k6vvBjGo
Okreglak Nat Metab 2023 video 3	https://youtu.be/Nj6VKtvCs7Q
Okreglak Nat Metab 2023 video 4	https://youtu.be/qEYoo35iYb8
Okreglak Nat Metab 2023 video 5	https://youtu.be/P-Px1AHvvE0
Okreglak Nat Metab 2023 video 6	https://youtu.be/LnHIm79F8m0
Okreglak Nat Metab 2023 video 7	https://youtu.be/SpozM5PJav4
Okreglak Nat Metab 2023 video 8	https://youtu.be/LW1ldhxYdUQ
Okreglak Nat Metab 2023 video 9	https://youtu.be/e226Dhs3hd0
Okreglak Nat Metab 2023 video 10	https://youtu.be/5rDD-3Aop7s
Okreglak Nat Metab 2023 video 11	https://youtu.be/pkQ4fgK3V5A
Okreglak Nat Metab 2023 video 12	https://youtu.be/PaljMXiAC2M
Okreglak Nat Metab 2023 video 13	https://youtu.be/SVlXEyiwwdM
