# Supplementary figures and images for: Cell cycle-linked vacuolar pH dynamics regulate amino acid homeostasis and cell growth
Source: Nat Metab. 2023 Aug 28;5(10):1803–19. doi: 10.1038/s42255-023-00872-1 (PMC10590757; doi:10.1038/s42255-023-00872-1)

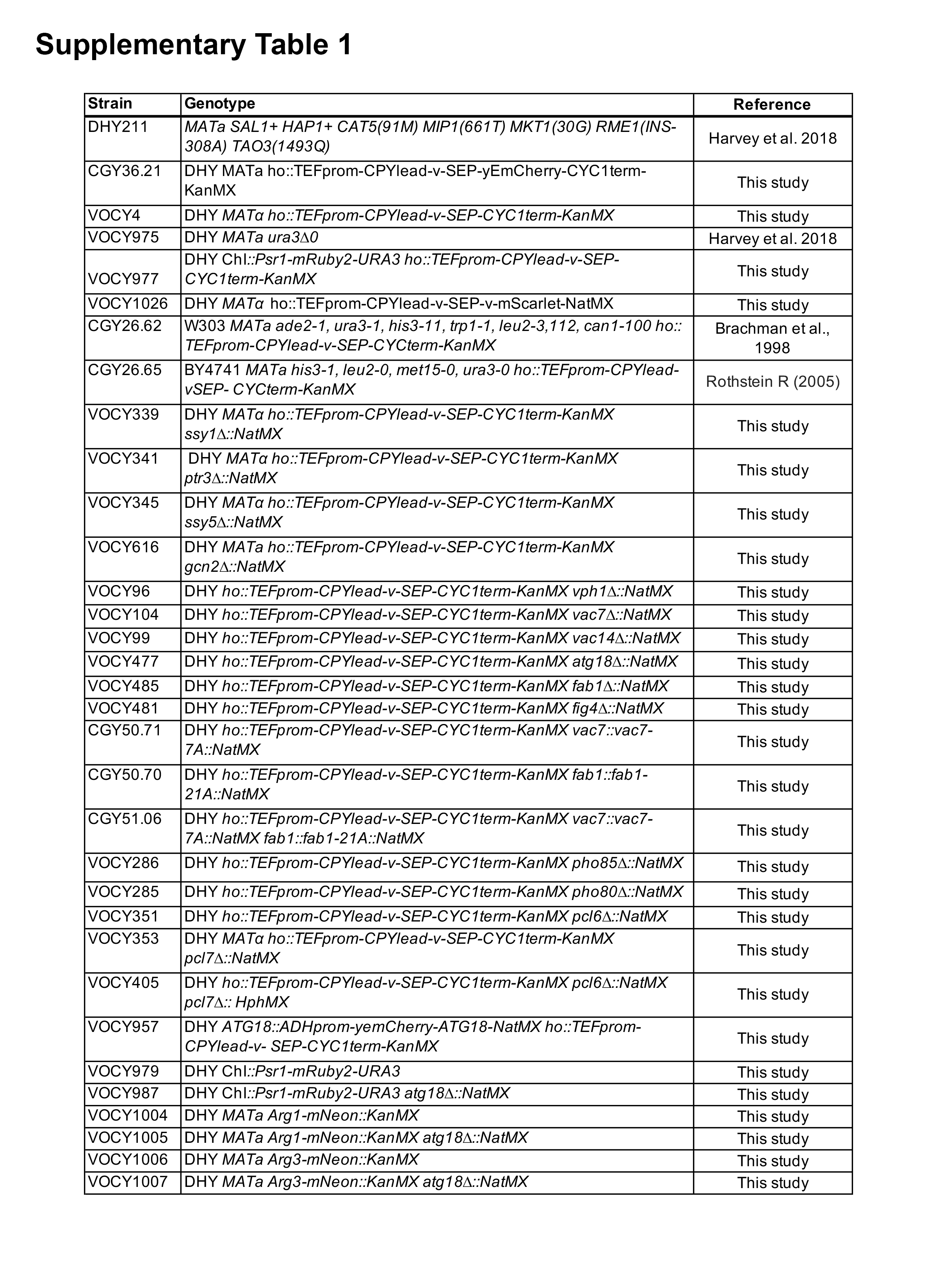

Supplement: Supplementary file 5 — Strains used in this study. [file 42255_2023_872_MOESM5_ESM.jpg]

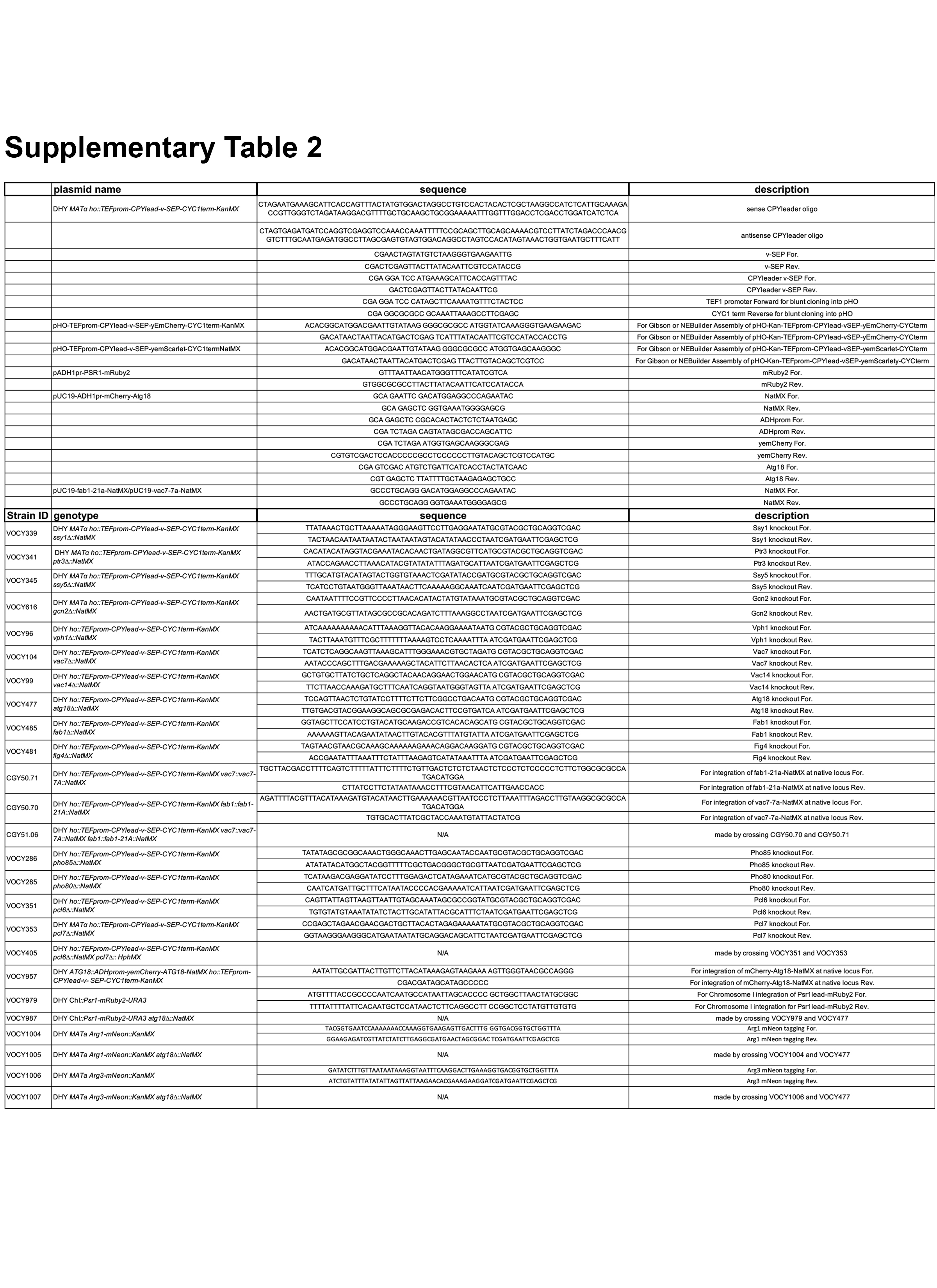

Supplement: Supplementary file 6 — Oligonucleotides used in this study. [file 42255_2023_872_MOESM6_ESM.jpg]
